# DPYD genotyping in patients receiving capecitabine: an exploratory analysis from the D-TORCH study

**DOI:** 10.3389/fphar.2026.1732128

**Published:** 2026-02-20

**Authors:** Hemavathi Baskarane, Mohit Kumar Divakar, Akhil P. Santhosh, Kriti Pallavi, Vishakha Hooda, Vamsi Krishna Yenamandra, Saran Kumar, Vrajesh Shetty, Jahnavi Banotra, Ishaan Gupta, Shilpi Minocha, Pranay Tanwar, Atul Sharma, Payal Vasudeva, Akash Kumar, R. Chethan, Sameer Bakhshi, Atul Batra

**Affiliations:** 1 Department of Medical Oncology, All India Institute of Medical Science, New Delhi, India; 2 Indian Institute of Science, Bengaluru, India; 3 Indian Institute of Technology, Delhi, India; 4 National Cancer Institute, Jhajjar, India

**Keywords:** cancer genomics, capecitabine toxicity, DPYD gene polymorphism, D-TORCH trial, pharmacogenomics (PGx), pharmacogentics

## Abstract

**Introduction:** Deficiency of the dihydropyrimidine dehydrogenase enzyme can result in capecitabine-related toxicity due to genetic alterations in the DPYD gene, leading to complete or partial DPD deficiency and poor or intermediate metabolizer phenotypes. The distribution of DPYD variants varies across populations. While routine DPYD genotyping is recommended in Western populations, data from India, particularly from next-generation sequencing (NGS)–based studies, remain limited.

**Methods:** This exploratory analysis was conducted within the D-TORCH trial, a randomized, double-blind, placebo-controlled study evaluating topical diclofenac for prevention of capecitabine-induced hand–foot syndrome. Germline whole-exome sequencing was performed in consenting patients prior to capecitabine initiation. DPYD variants were identified using an NGS pipeline, annotated via ANNOVAR and PharmGKB, and classified according to CPIC guidelines.

**Results: **Seventy-six patients underwent DPYD sequencing; 54 (71%) carried at least one variant and 22 (29%) were wild-type. Thirteen coding or splice-site variants were identified, including three (3.9%) associated with an intermediate metabolizer phenotype; no poor metabolizers were detected. The most common variants were classified as normal metabolizers. Grade 2 toxicity occurred in 63.6% of variant carriers and 55.6% of wild-type patients, with diarrhea and mucositis being most frequent. No significant association was observed between DPYD variant status and toxicity.

**Discussion:** This first NGS-based DPYD report from India highlights the low prevalence of clinically actionable variants. Larger studies are required to validate these findings and guide population-specific fluoropyrimidine dosing strategies.

## Background

Fluoropyrimidines, such as 5-fluorouracil (5-FU) and capecitabine, are widely used chemotherapeutic agents in the treatment of gastrointestinal, head and neck, and breast cancers (Masuda et al., 2017; Bang et al., 2012; Cassidy et al., 2008; Grothey et al., 2018; Geyer et al., 2006). These drugs are known to cause early-onset severe or life-threatening toxicities, including myelosuppression, mucositis, hand-foot syndrome, and diarrhea, in about 10 to 30% of patients, with reported mortality rates ranging from 0.5% to 1%] (Van Cutsem et al., 2001; Amstutz et al., 2009; Boisdron-Celle et al., 2017; Froehlich et al., 2015; Meulendijks et al., 2015; Lévy et al., 1998; Bajetta et al., 2005; Tsalic et al., 2003). The enzyme dihydropyrimidine dehydrogenase (DPD), which is coded by the DPYD gene, is the rate-limiting enzyme involved in the 5-FU metabolism (Thorn et al., 2011). Partial or complete deficiency of DPD activity is associated with life-threatening side effects among patients undergoing 5–FU–based treatment (Morel et al., 2006; Lunenburg et al., 2020). More than 450 DPYD polymorphisms have been associated with fluoropyrimidine-induced toxicity, and the incidence of DPD deficiency has been estimated at 3%–7% in the Caucasian population (Morel et al., 2006; Lunenburg et al., 2020; Amstutz et al., 2018).

Routine testing for four DPYD variants that are commonly found–DPYD2A (c.1905+1G>A), DPYD13 (c.1679T>G), c.2846A>T, and c.1129-5923C>G/hapB3 is recommended by the European Medicines Agency before the initiation of 5-FU chemotherapy (Lunenburg et al., 2020). These mutations play a role in the interindividual variability of 5-FU toxicity. The current guidelines of the Clinical Pharmacogenetics Implementation Consortium (CPIC) include 82 known DPYD variants, of which 21 are classified as poor metabolizers (no DPYD enzyme function), six as intermediate metabolizers (reduced function), and the rest as normal metabolizers or with limited evidence (Amstutz et al., 2018). The CPIC guidelines suggest avoiding 5-FU in poor metabolizers and a 25%–50 % dose reduction in intermediate metabolizers. (Lunenburg et al., 2020; Amstutz et al., 2018) While fluoropyrimidine toxicity profiles are comparable between populations, the prevalence and distribution of DPYD variants differ (White et al., 2021). Certain variants which are common in Europeans have not been reported in the African, Chinese, or Japanese populations, whilst other clinically relevant variants may be unique to non-Caucasians. Routine DPYD genotyping is not commonly practiced in India, and information on DPYD polymorphisms in the Indian population is scarce. Previously published data from India utilized PCR or Sanger 4 sequencing to examine known polymorphisms in most cases, including healthy volunteers or patients with grade 3 toxicities (Hariprakash et al., 2018; Patil et al., 2016).

To address these gaps, we conducted a single-center study using next-generation sequencing to evaluate the prevalence of DPYD variants in capecitabine-treated patients and their associated toxicity profiles.

## Methods

### Study design and patient cohort

This exploratory analysis was conducted in patients enrolled in the D-TORCH study, a phase III trial evaluating the efficacy of diclofenac gel in preventing capecitabine-induced hand-foot syndrome (HFS). D-TORCH was an investigator-initiated, single-center, randomized, double-blind, placebo-controlled trial conducted at the All-India Institute of Medical Sciences (AIIMS), New Delhi, between January 2021 and February 2023. The trial was prospectively registered with the Clinical Trials Registry of India (CTRI/202101/030592), approved by the Institutional Ethics Committee (IECPG-82/27.01.2021), and conducted in accordance with Indian Council of Medical Research (ICMR) guidelines and Good Clinical Practice (GCP) standards. The study protocol and primary results have been previously published (Santhosh et al., 2022)

Briefly, eligible patients were randomly assigned to receive either topical 1% diclofenac gel or a placebo for a 12-week study period. Overall toxicity was assessed using the Common Terminology Criteria for Adverse Events (CTCAE) during the first 12 weeks of treatment, with evaluations performed every 3-week cycle, and toxicity data have been previously reported in the D-TORCH trial. The gel was applied to both hands concurrently with oral capecitabine administration. The primary endpoint was the incidence of grade 2 or higher HFS. In patients willing to participate in the optional genetic study, a 5 mL blood sample was collected before therapy initiation for germline whole-exome sequencing (WES). In the current exploratory analysis, we present the analysis of *DPYD* gene polymorphisms.

### DNA extraction and sequencing

Genomic DNA was extracted from the blood samples using the QIAamp DNA mini kit (Cat. No. 51306, QIAGEN). Samples with a minimum concentration of 25 ng/ul were sent for DNA sequencing. DNA sequencing libraries were prepared through enzymatic fragmentation and ligation-based methods, per the manufacturer’s protocol (zGenTM DNA Library Prep EZ). Further, the libraries were subjected to capture and multiplexing as per the IDT capture protocol (xGen Hyb Panel v2). Post quality-check, the prepared library was sequenced on the Illumina NovaSeq 6000 platform with 150 × 2 base paired-end reads.

### Data analysis and variant calling

Sequencing data analysis was performed on the DRAGEN v4.2.4 pipeline on the DRAGEN Bio-IT platform, using the GRCh38 reference genome. Alignment and Post processing were followed by variant calling, and the variant file was generated in the VCF format. Next, a multi-sample VCF was generated for the cohort-wise analysis, having only the variants with the “PASS” filter. The generated multi-sample VCF was further annotated via the ANNOVAR annotation tool. Further, the annotated VCF was filtered for the exonic variants and analysed for their functional relevance related to the capecitabine toxicity. We also referred to the PharmGKB database for individual variants, which contains the clinical variant annotation CPIC guidelines for the genetic variants’ response to different medications.

### Statistical analysis

Participants’ baseline clinical characteristics and toxicity profiles were presented using descriptive statistics, including median (inter-quartile range), mean (standard deviation) and frequencies. No inferential statistical tests were performed. All statistical analysis was done using SPSS version 29.0 (IBM Corp. Released 2023. IBM SPSS Statistics for Windows, Version 29.0.2.0 Armonk, NY: IBM Corp). Firth-adjusted logistic regression and stratified sensitivity analyses were performed using R version 4.3.1 (R Foundation for Statistical Computing, Vienna, Austria), employing the logistf package A lollipop plot depicting the distribution of DPYD variants was generated using the Mutation Mapper tool on cBioportal.

## Results

Among the 263 patients in the D-TORCH study, *DPYD* genotyping was performed in 76 patients, who agreed to participate in this exploratory sub-study (Supplementary Figure S1). Baseline characteristics such as age, sex, cancer type, prior chemotherapy exposure, regimen type, and randomized arm were similar between the sequenced subgroup and the whole cohort, although a higher proportion of patients in the sequenced cohort had metastatic disease (36.4% vs. 21.4%, P = 0.007. However, with respect to toxicity, patients in the sequenced cohort experienced a higher, statistically significant proportion of grade 2–3 diarrhea, whereas the frequencies of other treatment-related toxicities were comparable between the groups. Of these, 54 (71%) harbored at least one *DPYD* variant, while 22 (29%) were wild-type. The median age of the participants was 47 years (range: 27–71), with a female predominance (69.7%). Most patients had an ECOG performance status of 0–1 (55.3%), and breast and gastrointestinal malignancies were similarly distributed. Most patients had metastatic breast or gastrointestinal cancer. Baseline characteristics and toxicity profiles of the sequenced subgroup versus the whole cohort are summarized in Table 1. Comparisons between DPYD variant-positive and wild-type patients are detailed in Table 2, and no statistically significant differences were observed in baseline characteristics between the variant-positive and variant-negative groups or by the metabolizer phenotype (Supplementary Table S1).

**TABLE 1:** Comparison of baseline characteristics and toxicity profile between the sequenced subgroup and the whole cohort.

Variable	DPYD not tested (n = 187)	DPYD tested (n = 76)	P value
Age (years), mean ± SD	47.1 ± 11.6	48.2 ± 11.6	0.484
Sex	​	​	0.755
Male	53 (28.3%)	23 (30.3%)	​
Female	134 (71.7%)	53 (69.7%)	​
Drug/Placebo arm	​	​	0.350
Drug	98 (52.4%)	35 (46.1%)	​
Placebo	89 (47.6%)	41 (53.9%)	​
Regimen type	​	​	0.046
Monotherapy	84 (44.9%)	24 (31.6%)	​
Combination therapy	103 (55.1%)	52 (68.4%)	​
Cancer type	​	​	0.191
Breast	77 (41.2%)	38 (50.0%)	​
Gastrointestinal	110 (58.8%)	38 (50.0%)	​
Prior chemotherapy	​	​	0.060
No	124 (66.3%)	41 (53.9%)	​
Yes	63 (33.7%)	35 (46.1%)	​
Stage	​	​	​
Curative	103 (78.6%)	28 (21.4%)	0.007
Palliative	84 (63.6%)	48 (36.4%)	​
Toxicity profile ((≥ grade 2)			
Diarrhea	35/187 (18.7%)	30/76 (39.5%)	<0.001
Mucositis	41/187 (21.9%)	25/76 (32.9%)	0.063
Myelosuppression*	6/187 (3.2%)	3/76 (3.9%)	0.721
HFS grade 2–4*	17/187 (9.1%)	8/76 (10.5%)	​

*P* values were calculated using the Wilcoxon rank-sum test for age.

χ^2^ test for sex, drug/placebo arm, regimen type, cancer type, prior chemotherapy, stage, diarrhea (≥ Grade 2), and mucositis (≥ Grade 2).

*- Fisher’s exact test was applied.

All statistical tests were two-sided.

**TABLE 2:** Baseline characteristics by variant status (N = 76).

Total	All patients N = 76 (%)	DPYD variant N = 54 (%)	Wild type variant N = 22 (%)	P value
Age (mean ± SD)	48.2 ± 11.6	48.59 ± 12.07	46.09 ± 10.34	0.39
Sex Male Female	23 (30.3) 53 (69.7)	17 (31.5%) 27 (68.5%)	6 (27.3) 16 (72.7)	0.789
PS 0–1 2	42 (55.3) 34 (44.7)	32 (59.3) 22 (40.7)	10 (45.5) 12 (54.5)	0.316
Randomized arm Diclofenac Placebo	41 (53.9) 35 (46.1)	26 (48) 28 (52)	15 (68.2) 7 (31.8)	0.11
Type of cancer Breast Gi	38 (50) 38 (50)	26 (48) 28 (52)	12 (54.5) 10 (45.5)	0.801
Curative Palliative	28 (36.8) 48 (63.2)	20 (37) 34 (63)	8 (36.4) 14 (63.6)	1.00
Mono Combination therapy	24 (31.6) 52 (68.4	15 (28) 39 (72)	9 (40.9) 13 (59.1)	0.287
Previous chemotherapy received	35 (46.1)	26 (48)	9 (40.9)	0.619
BSA BMI	1.53 (1.33–1.77) 21.3 (15.6–28.4)	1.53 (1.38–1.77) 21.4 (18–28)	1.53 (1.33–1.75) 21.1 (15.6–28.4)	--
Dose of capecitabine, mg/m2, mean (SD)	2901 (383)	2907.4 (364)	2886.3 (434)	0.83
Dose reduction at any time	21	17	4	0.24

### Variant spectrum of the DPYD gene in the patient cohort

In the cohort of 76 patients sequenced, we identified 123 high-quality genetic variants in the DPYD gene. Sequencing data was also assessed on various parameters to check the overall sequencing data consistency. This yielded an average sequencing depth of around 203.84x and a callable fraction ranging from 0.86 to 0.99 among all the samples (Supplementary Table S2). Detailed sequencing data along with per-exon coverage and read depth per sample are listed in Supplementary Table S3; Supplementary Table S4. Moreover, uniform VCF-level filters included mapping quality (MQ) and overall quality (QUAL), such that variants with mapping quality less than 40 (MQ < 40) and over-all QUAL less than 30 (QUAL<30) were filtered out at VCF level and only the variants that pass all filters and labelled as “PASS” in VCF were retained. This yielded a total of 123 genetic variants, of which, 10 multiallelic variants were not covered in this analysis (Supplementary Table S5), since they were present as repeat elements and require a separate set of informatic tools and annotations. Therefore, the present study covers the 113 single nucleotide polymorphisms (SNPs) and Insertions/Deletions (InDels) analyzed. There were 104 SNPs and 9 InDels, which were further filtered to prioritise only exonic and splicing variants. This resulted in a total of 13 variants in the coding region, comprising 12 exonic variants and one splicing variant (Table 3; Supplementary Figure S2). Of the 12 exonic variants, nine were non-synonymous and three were synonymous. The most frequently observed variants were rs1801265 (n = 25), rs1801159 (n = 12), and rs1801160 (n = 13). The distribution of these variants varied, with 33 patients (61%) harboring a single variant, 13 patients (24%) carrying two variants, and eight patients (15%) carrying three variants.

**TABLE 3:** List of exonic variants identified in our cohort.

Chr	Start	End	Ref	Alt	Func.refGene	Gene.refGene	GeneDetail.refGene	ExonicFunc.refGene	AAChange.refGene	avsnp151
chr1	97305279	97305279	G	A	Exonic	DPYD	.	Nonsynonymous SNV	DPYD:NM_000110:exon18:c.C2279T:p.T760I	rs112766203
chr1	97305364	97305364	C	T	Exonic	DPYD	.	Nonsynonymous SNV	DPYD:NM_000110:exon18:c.G2194A:p.V732I	rs1801160
chr1	97450068	97450068	A	G	Exonic	DPYD	.	Synonymous SNV	DPYD:NM_000110:exon14:c.T1896C:p.F632F	rs17376848
chr1	97515839	97515839	T	C	Exonic	DPYD	.	Nonsynonymous SNV	DPYD:NM_000110:exon13:c.A1627G:p.I543V	rs1801159
chr1	97515865	97515865	C	T	Exonic	DPYD	.	Nonsynonymous SNV	DPYD:NM_000110:exon13:c.G1601A:p.S534N	rs1801158
chr1	97515921	97515921	A	C	Exonic	DPYD	.	Synonymous SNV	DPYD:NM_000110:exon13:c.T1545G:p.V515V	​
chr1	97515923	97515923	C	T	Exonic	DPYD	.	Nonsynonymous SNV	DPYD:NM_000110:exon13:c.G1543A:p.V515I	rs148994843
chr1	97573863	97573863	C	T	Exonic	DPYD	.	Synonymous SNV	DPYD:NM_000110:exon11:c.G1236A:p.E412E	rs56038477
chr1	97679170	97679170	T	C	Exonic	DPYD	.	Nonsynonymous SNV	DPYD:NM_000110:exon8:c.A775G:p.K259E	rs45589337
chr1	97699535	97699535	T	C	Exonic	DPYD	.	Nonsynonymous SNV	DPYD:NM_000110:exon6:c.A496G:p.M166V	rs2297595
chr1	97740426	97740426	T	A	Exonic	DPYD	.	Nonsynonymous SNV	DPYD:NM_000110:exon4:c.A287T:p.D96 V,DPYD:NM_001160301:exon4:c.A287T:p.D96V	rs771573678
chr1	97883329	97883329	A	G	Exonic	DPYD	.	Nonsynonymous SNV	DPYD:NM_000110:exon2:c.T85C:p.C29R,DPYD:NM_001160301:exon2:c.T85C:p.C29R	rs1801265
chr1	97450058	97450058	C	T	Splicing	DPYD	NM_000110:exon14:c.1905 + 1G>A	​	​	rs3918290

### Drug response/toxicity related variants

For understanding the clinical relevance of the 13 coding variants, we utilised the PharmgKB database with CPIC guidelines for capecitabine and the DPYD gene. Of the 13 variants, nine were assigned as Level 1A, in terms of evidence available for the variant-specific guidelines. Level 1A variants are those with the highest clinical annotation levels of evidence for the variant-drug prescribing guidelines. Of the 9 Level 1A variants, two variants had altered DPD activity. We also mapped the observed genotype and individual variant alleles for all the 13 variants, to their assigned enzyme activity value and calculated the enzyme activity score for the respective genotypes. Then, an annotated metabolizer phenotype was listed for the respective variant. This showed three variants with altered DPD activity (Supplementary Table S6).

First variant rs56038477 (DPYD:NM_000110:exon11:c.G1236A:p.E412E) was present in heterozygous condition such that the mutated allele had an assigned activity value of 0.5 and the wild type allele having 1.0 activity value, such that a calculated activity score for this genotype was 1.5. This allele had an Intermediate metabolizer phenotype (1.5) reported, implicated in a decreased DPD gene activity (30%–70% of normal), and an increased risk for drug toxicity for fluoropyrimidine drugs. This variant is a synonymous variant, in which the T allele represents a marker for the HapB3 haplotype allele (an intronic variant) and an associated decreased function as per the CPIC guidelines.

The second variant identified (rs3918290) was a splicing variant in the DPYD gene (DPYD:NM_000110:c.1905 + 1G>A) being a heterozygous variant with an assigned activity value of 0.0 for mutated allele and 1.0 activity value for the wild type allele, such that a calculated activity score for this genotype was 1.0. Therefore resulting in an Intermediate metabolizer phenotype (1.0), a decreased DPD activity (30%–70% of normal), and an increased risk for fluoropyrimidine drug toxicity.

We also identified a third variant, rs112766203, which was not assigned to any level of evidence (such as Level 1A, 1B), but has the variant annotations as per the CPIC guidelines for capecitabine and DPYD. This exonic variant rs112766203 (DPYD:NM_000110:exon18:c.C2279T:p.T760I) was also present in heterozygous condition such that the mutated allele had an assigned activity value of 0.5 and the wild type allele having 1.0 activity value, such that a calculated activity score for this genotype was 1.5, which results in a phenotype of intermediate metabolizer (1.5), a decreased DPD activity (30%–70% of normal), and an increased risk for fluoropyrimidine drug toxicity.

In summary, we identified three variants resulting in intermediate metabolizer phenotype and a reduced DPD gene activity in our cohort (Supplementary Figure S3). We also identified two more variants, for which no information is available as per the CPIC guidelines or PharmGKB database, but predicted to be the Variant of Unknown Significance (VUS) from the Franklin automated ACMG classification.

### Correlation of toxicity and mutations

Among DPYD variant carriers (n = 54), 66.7% (36 patients) experienced Grade 2 or 3 toxicity, comparable to 63.6% (14 patients) in the DPYD wild-type group (n = 22) (Table 4). Diarrhea was the most common toxicity in the DPYD variant group, affecting 40.8% (22 patients), with 25.9% (n = 14) experiencing grade 2 and 13% (n = 7) experiencing Grade 3 toxicity. Mucositis was observed in an equal proportion (40.8%, 22 patients), including 35.2% (19 patients) with Grade 2 and 3.7% (2 patients) with grade 3 toxicity. HFS occurred in 13% (7 patients ), with Grade 2 in 9.3% (5 patients) and Grade 3 in 1.9% (1 patient), while hematological toxicity (Grade 2) was noted in 3.7% (2 patients). A similar toxicity pattern was seen in DPYD wild-type patients, with diarrhea in 45.4% (10 patients) and mucositis in 31.7% (7 patients). HFS occurred in 13.5% (3 patients), while hematological toxicity (Grade 2) was reported in 4.5% (1 patient). Although both mucositis and diarrhea were numerically higher in the DPYD variant cohort, odds ratios indicated no statistically significant differences between variant and wild-type patients for any toxicity outcome (diarrhea ≥ grade 2 OR 0.65, 95% CI 0.24–1.78, p = 0.443; overall grade ≥2 toxicity OR 0.71, 95% CI 0.26–1.98, p = 0.612) (Table 4). Among the three Intermediate metabolizers, grade 3 diarrhoea was observed in one patient with rs3918290, while no toxicity was observed in variants rs112766203 and rs56038477. A patient with a VUS and a novel variant did not report any adverse events.

**TABLE 4:** Correlation of toxicity and mutations observed in patients.

Toxicity	Variant (n = 22)	Wild-type (n = 54)	Odds ratio	95% CI	P value
Diarrhoea ≥ grade 2	10 (45.5%)	19 (35.2%)	0.65	0.24–1.78	0.443
Diarrhoea (any grade)	10 (45.5%)	20 (37.0%)	0.71	0.26–1.93	0.606
Mucositis (any grade)	9 (40.9%)	20 (37.0%)	0.85	0.31–2.34	0.798
Mucositis ≥ grade 2	9 (40.9%)	18 (33.3%)	0.72	0.26–2.00	0.601
Myelosuppression (any/≥G2)	1 (4.5%)	2 (3.7%)	0.81	0.07–9.39	1.000
Overall toxicity (any grade)	14 (63.6%)	32 (59.3%)	0.83	0.30–2.32	0.800
Overall toxicity ≥ grade 2	14 (63.6%)	30 (55.6%)	0.71	0.26–1.98	0.612

Among normal metabolizers (n = 50), 70% (35) experienced grade 2 or 3 toxicity. Mucositis was reported in 42% (21 patients), with grade 2 in 38% (19 patients) and grade 3 in 4% (2 patients). Diarrhea occurred in 40% (20 patients), with grade 2 in 28% (14 patients) and grade 3 in 12% (6 patients). HFS was observed in 12% (6 patients), with grade 2 in 10% (5 patients) and grade 3 in 2% (1 patient). (Supplementary Table S7) Furthermore, variant-specific toxicities among all the patients screened are also detailed in the Supplementary Table S8 for the grade 3 or higher toxicities. Comparisons between intermediate and normal metabolizers should be interpreted cautiously given the limited sample size of intermediate metabolizers. As a result, several endpoints yielded non-estimable (NE) estimates with wide confidence intervals, limiting inference on toxicity differences by metabolizer status (Supplementary Table S9). To address the limited sample size, adjusted analyses were conducted using Firth penalized logistic regression. Separate models were fitted for grade ≥2 hand–foot syndrome (HFS), grade ≥2 diarrhoea, grade ≥2 mucositis, and any grade ≥2 toxicity, adjusting for age, sex, cancer type, prior chemotherapy exposure, treatment modality (monotherapy vs. combination), randomized arm, mean capecitabine dose received, and DPYD variant status. In all models, none of the covariates were significantly associated with the occurrence of grade ≥2 toxicities (Table 5).

**TABLE 5:** Multivariable firth logistic regression for grade ≥2 toxicities.

Variable	Grade ≥2 HFS OR (95% CI)	*P* value	Grade ≥2 diarrhoea OR (95% CI)	*P* value	Grade ≥2 mucositis OR (95% CI)	*P* value	Any ≥ grade 2 toxicity OR (95% CI)	*P* value
Model N (events)	N = 76 (8)	​	N = 76 (29)	​	N = 76 (27)	​	N = 76 (30)	​
Age	1.04 (0.98–1.12)	0.22	1.02 (0.98–1.06)	0.44	1.01 (0.97–1.06)	0.49	1.01 (0.97–1.06)	0.56
Sex (female vs. male)	3.42 (0.36–40.4)	0.28	0.95 (0.22–3.95)	0.94	0.56 (0.13–2.21)	0.41	0.44 (0.10–1.91)	0.27
Cancer type (GI vs. breast)	2.51 (0.21–30.0)	0.44	0.41 (0.06–2.16)	0.30	1.25 (0.23–6.45)	0.79	0.73 (0.14–3.85)	0.70
Prior chemotherapy (yes vs. no)	2.01 (0.24–20.6)	0.51	0.40 (0.09–1.56)	0.19	0.94 (0.25–3.57)	0.92	0.68 (0.17–2.66)	0.58
Combination vs. monotherapy	0.75 (0.13–4.40)	0.74	1.73 (0.55–6.21)	0.36	0.87 (0.28–2.74)	0.81	1.15 (0.37–3.49)	0.81
Randomized arm (intervention vs. control)	2.93 (0.65–16.8)	0.17	1.00 (0.38–2.62)	0.99	0.75 (0.27–1.98)	0.56	0.85 (0.31–2.28)	0.74
Mean capecitabine dose received	1.00 (0.99–1.00)	0.12	1.00 (0.99–1.00)	0.37	1.00 (0.99–1.00)	0.74	1.00 (0.99–1.00)	0.84
DPYD variant (yes vs. no)	0.69 (0.14–4.12)	0.66	0.69 (0.25–1.89)	0.46	0.74 (0.27–2.06)	0.55	0.52 (0.16–1.51)	0.23

Odds ratios and 95% confidence intervals were estimated using Firth’s penalized logistic regression in R with the logistf package.

Sensitivity analyses were performed for HFS toxicity alone, as topical diclofenac has minimal systemic absorption and is not expected to affect other toxicities. The DPYD genotype by treatment arm interaction showed no evidence of effect modification (any grade OR 2.26, 95% CI 0.11–60.57, p = 0.596; grade ≥2 OR 0.87, 95% CI 0.03–26.39, p = 0.931), indicating that diclofenac did not confound the relationship between DPYD genotype and HFS.

## Discussion

In this exploratory sub-study of the D-TORCH trial, 54 of the 76 sequenced patients (71%) harboured at least one DPYD variant, while no poor metabolizers were detected.; however, three individuals were determined to be intermediate metabolizers as per the activity values for the identified genotype, one of whom developed grade 3 toxicity. The remaining 51 individuals were classified as normal metabolizers, including one who harbored an unknown variant (no RSID) and showed no adverse effects. Among the four DPYD variants recommended by the European Medical Agency (EMA) for routine testing, rs3918290 has been the most frequently reported variant in previous Indian studies in association with grade 3 toxicity (Pavithran et al., 2021; Chan et al., 2024). However, in our cohort, only one patient carried this variant. This discrepancy is presumably due to prior studies focusing solely on patients with grade 3 toxicities, whereas we carried out NGS regardless of clinical toxicity.

The most prevalent variants found among the normal metabolizers in our study (n = 50) were rs1801265 (n = 25), rs1801159 (n = 12), rs1801160 (n = 13), and rs2297595 (n = 7). Based on CPIC guidelines, these variants do not require dose adjustment. Nevertheless, their clinical relevance is uncertain, with conflicting evidence across various ethnic populations. Interestingly, 35 out of 50 patients in our cohort, classified as normal metabolizer phenotype as per the CPIC guidelines, still developed grade 2 or 3 toxicity, emphasizing the limitation of CPIC-based phenotype prediction and dose adjustment in the Indian population. Our findings align with previous Indian studies. Patil et al. selectively tested 12 Indian patients with grade 3 toxicities during TPF (Docetaxel,5-fluorouracil (5-FU), and cisplatin) neoadjuvant therapy and reported normal metabolizers in rs1801160, rs1801265, or rs2297595 in 11 patients (Patil et al., 2016). Similarly, Sahu et al. conducted PCR-based testing in 22 gastrointestinal cancer patients with grade 3 toxicities, detecting normal metabolizer phenotypes in rs1801265 (n = 8), rs2297595 (n = 4), and rs1801160 (n = 4) (Sahu et al., 2016). Furthermore, a South Indian study of 375 patients employing PCR-based testing for four variants identified rs2297595 in 32 of 47 DPYD-mutated individuals, tying it to fluoropyrimidine toxicity (Pavithran et al., 2021). Another study using Sanger sequencing of selective variants in 1064 Indian patients receiving fluoropyrimidines reported a DPYD variant frequency of 27.2 percent (289/1064), involving either homozygous or heterozygous mutations in rs1801160 and rs1801159, though data on clinical toxicity was not described (Patil et al., 2019).

In addition, two population-based studies of DPYD polymorphisms in Indians also highlighted genetic variability. Hariprakash et al. analyzed almost 3,000 South Asians, validating their findings in 110 Indian cancer patients, and observed that certain variants were comparable to those found in individuals of European ancestry, while others were more closely related to those seen in individuals of African origin (Hariprakash et al., 2018). Heterozygous variants rs1801265, rs1801159, rs1801160, rs2297595, and rs12022243 were frequent in the general population. Notably, rs2297595, despite being a standard metabolizer variant, was associated with toxicity in the cancer cohort. The second population-based study evaluated 2000 Indian individuals, mapping DPYD variant frequencies and their functional activity using bioinformatic tools. Deleterious variants were found at a frequency of 1.89%, while normal metabolizers per CPIC were at a frequency of 58.3% in rs1801265, rs2297595, rs1801160, rs75017182, and rs1801158. In silico/CUP-SAT analyses predicted an impaired DPD enzyme nature for rs1801160 and rs1801158 but not for rs2297595, in contrast to the earlier study. Population clustering analysis of Indians was in line with South Asians being distinct from Europeans and Africans, underscoring ethnic variations in DPYD polymorphisms and 5-FU toxicity risk (Naushad et al., 2021).

In the recent systematic review by Chan et al., conducted exclusively in non-European ancestry, a higher frequency of novel DPYD variants was observed, along with an association between the normal metabolizer phenotype and fluoropyrimidine toxicity, despite overall toxicity rates being similar to those in Caucasians (Chan et al., 2024). In our study, we identified a variant of uncertain significance and a previously unreported novel variant. Similarly, Lopes et al., found that around 6% of the patient population (n = 631) had a DPYD variant that was overlooked by targeted genotyping (López López-Cepero et al., 2023). These included functionally important intronic variants within haplotype B3, which evince the limitations of conventional screening methods. NGS may better capture rare or intronic variants over PCR, though targeted panels with intronic coverage remain a viable and cost-effective alternative. Among 22 patients with DPYD wild-type, 13 experienced grade 2/3 toxicity, hinting the role of additional enzyme polymorphisms such as thymidine synthase and MTHFR involved in the 5-FU metabolism (Loganayagam et al., 2013). Routine sequencing may inform toxicity prediction by mapping gene polymorphisms that contribute to toxicity and remain uncharacterized in the Indian context.

Ethnic diversity also highlights the importance of genotype-phenotype association in decoding DPD enzyme function (White et al., 2021). The DPYD activity score, validated in the Caucasian population, remains unverified in other ethnic groups, potentially explaining toxicity differences among CPIC-classified normal metabolizers (White et al., 2021). Limited genotype-phenotype studies have also identified population-specific variations, such as the 1905 + 1G>A intronic variant, which is prevalent in Iranians but does not mirror the same toxicity profile seen in Europeans (Negarandeh et al., 2020).

Overall, in the present study, we identified exonic variants in the DPYD gene in 71% of patients (54 out of 76) in our cohort. Of these, 3.9% of patients (3 out of 76) had a DPYD exonic variant with a recommended intermediate metabolizer phenotype as per the CPIC guidelines. Further, we observed that some exonic variants detected in our cohort were also present in patients with grade 3 toxicity, however, inconsistent evidence exists for such variants. Additionally, there were patients with grade 3 or higher toxicity and no DPYD exonic variant observed. Such patients can be considered for further investigation of other underlying genetic mechanisms. Traditional PCR-based genotyping covers only targeted genetic variants and the broader gene-based variants are not captured in PCR-based assay. Our study represents the first report from India on DPYD gene-based investigation using NGS assay. However, it is limited by a smaller sample size and underrepresentation of patients with higher grade toxicity. In this context, these findings should be viewed primarily as an exploratory and descriptive assessment, rather than a definitive evaluation of the relationship between DPYD variants and clinical toxicity. Only 29% of the parent D-TORCH cohort underwent optional genotyping, and the intermediate metabolizer group was very small, limiting statistical power and producing wide confidence intervals. While baseline characteristics were broadly similar, the sequenced cohort included slightly more metastatic and combination-therapy patients and exhibited a higher proportion of ≥grade 2 diarrhea; however, no association was found with other toxicities. These factors support a cautious, hypothesis-generating interpretation of the findings. Moreover, our study has genetic analysis limited to exonic and splicing variants. Yet intronic DPYD risk alleles will not be captured directly, rather these would be tagged with exonic variants, such as exonic variants rs56038477 is often used as a tag SNP for the HapB3 haplotype. Also, in certain cases with deep intronic DPYD risk alleles, whole exome sequencing may not capture such intronic variants and true prevalence might be underreported. In this exploratory work, we also acknowledge the limitation that no orthogonal confirmation of clinically relevant calls such as rs56038477 was performed. Large-scale genotype-phenotype correlation studies are required to precisely define the impact of DPYD gene polymorphisms in the Indian population and their therapeutic implication.

## Conclusion

In summary, the present study demonstrates the primary insights into the capecitabine-related toxicity and DPYD exonic variants among Indian patients. Ethnic differences in the DPYD polymorphic variants exist in our population, underscoring the importance of phenotype correlation and routine NGS-based testing over PCR-based screening for enhanced toxicity prediction. Findings from our study lay the groundwork for further investigation on the capecitabine-related toxicity and DPYD exonic variants. Larger studies are needed to corroborate these findings and develop population-specific recommendations for safer use of fluoropyrimidines.

## Data Availability

The datasets presented in this article are not readily available due to ethical and legal restrictions, because sequencing data contains information that could compromise the participants' privacy. Requests to access such datasets should be directed to the corresponding author.
