# Supplementary material for: DPYD genotyping in patients receiving capecitabine: an exploratory analysis from the D-TORCH study
Source: Front Pharmacol. 2026 Feb 20;17:1732128. doi: 10.3389/fphar.2026.1732128 (PMC12963234; doi:10.3389/fphar.2026.1732128)
Supplement: Supplementary file 1 [file Supplementaryfile1.docx]

SUPPLEMENTARY:

NO OF PATIENTS RECRUITED IN DTORCH TRIAL N=263

EVALUABLE SAMPLES N=82

DPYD VARIANTS DETECTED

N=54

QC FAILURE

N=6

NEGATIVE

N=22

NOT TESTED N=181

(DENIED CONSENT )

**Supplementary figure 1: Flowchart depicting the DPYD genotyping in the D-TORCH trial.**


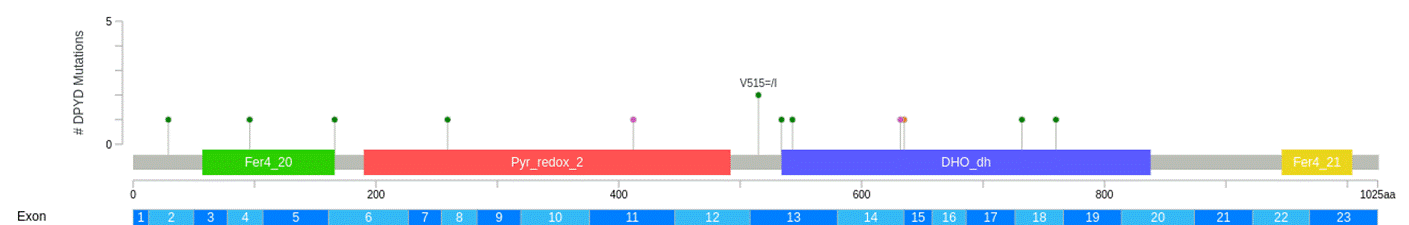


**Supplementary figure 2. Lollipop graph of all the exonic germline mutations identified in the entire cohort**


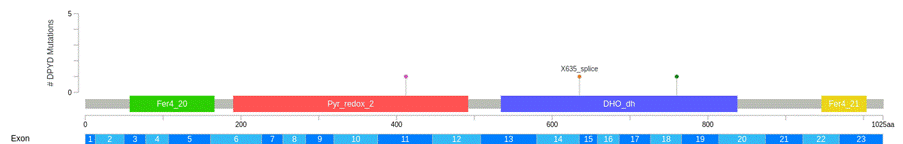


**Supplementary figure 3. Lollipop graph of the three exonic germline mutations with dosing guidelines available**

**Supplementary table 1:Baseline characteristics by metabolizer status (n = 53)**

| **Variable** | **Normal metabolizer (M, n = 50)** | **Intermediate metabolizer (P, n = 3)** | **P value** |
| --- | --- | --- | --- |
| Age (years), mean ± SD | 48.7 ± 12.2 | 50.0 ± 13.0 | 0.863 |
| Sex |  |  | 0.238 |
| Male | 15 (30%) | 2 (66.7%) |  |
| Female | 35 (70%) | 1 (33.3%) |  |
| Randomized arm |  |  | 0.610 |
| Diclofenac | 24 (48%) | 2 (66.7%) |  |
| Placebo | 26 (52%) | 1 (33.3%) |  |
| Treatment intent |  |  | 0.041 |
| Curative | 16 (32%) | 3 (100%) |  |
| Palliative | 34 (68%) | 0 (0%) |  |
| Therapy type |  |  | 0.557 |
| Monotherapy | 14 (28%) | 0 (0%) |  |
| Combination | 36 (72%) | 3 (100%) |  |
| Type of cancer |  |  | 0.238 |
| Breast | 25 (50%) | 0 (0%) |  |
| GI cancer | 25 (50%) | 3 (100%) |  |
| Prior chemotherapy | 26 (52%) | 0 (0%) | 0.236 |
| Capecitabine dose (mg/m²), mean ± SD | 2890.0 ± 368.3 | 3166.7 ± 288.7 | 0.209 |

**Supplementary table 2: Overall sequencing depth and callable fraction of the sequencing data for patients in our cohort.**

| **Sr. No.** | **Patient number/ID** | **Overall sequencing depth/Coverage** | **Callable_fraction** |
| --- | --- | --- | --- |
| 1 | 1323002960 | 221.84 | 0.94 |
| 2 | 1323002963 | 179.06 | 0.95 |
| 3 | 1323002965 | 162.53 | 0.87 |
| 4 | 1323002966 | 216.08 | 0.93 |
| 5 | 1323002967 | 237.84 | 0.94 |
| 6 | 1323002968 | 228.05 | 0.86 |
| 7 | 1323002969 | 337.5 | 0.94 |
| 8 | 1323002970 | 370.86 | 0.95 |
| 9 | 1323002971 | 518.09 | 0.96 |
| 10 | 1323002973 | 161.19 | 0.94 |
| 11 | 1323002974 | 103.32 | 0.98 |
| 12 | 1323002975 | 156.53 | 0.95 |
| 13 | 1323002978 | 224.61 | 0.97 |
| 14 | 1323002984 | 233.11 | 0.94 |
| 15 | 1323002987 | 186.12 | 0.93 |
| 16 | 1323002989 | 240.17 | 0.95 |
| 17 | 1323002991 | 340.9 | 0.92 |
| 18 | 1323002992 | 136.34 | 0.96 |
| 19 | 1323002993 | 269.28 | 0.95 |
| 20 | 1323002994 | 301.32 | 0.97 |
| 21 | 1323002996 | 192.97 | 0.96 |
| 22 | 1323002998 | 568.94 | 0.94 |
| 23 | 1323002999 | 103.63 | 0.93 |
| 24 | 1323003000 | 143.73 | 0.92 |
| 25 | 1323003001 | 249.01 | 0.94 |
| 26 | 1323003002 | 165.67 | 0.92 |
| 27 | 1323003003 | 223.72 | 0.95 |
| 28 | 1323003004 | 183.22 | 0.95 |
| 29 | 1323003005 | 166.46 | 0.95 |
| 30 | 1323003006 | 368.35 | 0.93 |
| 31 | 1323003008 | 160.6 | 0.92 |
| 32 | 1323003011 | 211.41 | 0.96 |
| 33 | 1323003012 | 292.81 | 0.97 |
| 34 | 1323003013 | 202 | 0.95 |
| 35 | 1323003014 | 232.39 | 0.95 |
| 36 | 1323003015 | 210.29 | 0.95 |
| 37 | 1323003016 | 318.3 | 0.91 |
| 38 | 1323003017 | 300.5 | 0.96 |
| 39 | 1323003018 | 403.84 | 0.95 |
| 40 | 1323003019 | 435.82 | 0.94 |
| 41 | 1323003020 | 230.53 | 0.96 |
| 42 | 1323003022 | 304.11 | 0.97 |
| 43 | 1323003023 | 235.73 | 0.97 |
| 44 | 1323003024 | 268.29 | 0.96 |
| 45 | 1323003025 | 300.85 | 0.94 |
| 46 | 1323003026 | 287.28 | 0.96 |
| 47 | 1323003027 | 156.122 | 0.93 |
| 48 | 1323003028 | 116.702 | 0.93 |
| 49 | 1323003029 | 86.75 | 0.98 |
| 50 | 1323003030 | 180.39 | 0.97 |
| 51 | 1323003031 | 96.8331 | 0.98 |
| 52 | 1323003032 | 93.182 | 0.95 |
| 53 | 1323003033 | 147.782 | 0.99 |
| 54 | 1323003034 | 157.674 | 0.95 |
| 55 | 1323003035 | 116.818 | 0.97 |
| 56 | 1323003036 | 119.179 | 0.92 |
| 57 | 1323003037 | 81.78 | 0.97 |
| 58 | 1323003038 | 180.277 | 0.96 |
| 59 | 1323003039 | 127.87 | 0.97 |
| 60 | 1323003040 | 140.65 | 0.95 |
| 61 | 1323003041 | 98.94 | 0.96 |
| 62 | 1323003042 | 146.477 | 0.96 |
| 63 | 1323003043 | 83.2642 | 0.98 |
| 64 | 1323003044 | 83.481 | 0.96 |
| 65 | 1323003046 | 125.463 | 0.96 |
| 66 | 1323003047 | 129.338 | 0.91 |
| 67 | 1323003048 | 95.13 | 0.95 |
| 68 | 1323003049 | 125.31 | 0.98 |
| 69 | 1323003050 | 90.44 | 0.92 |
| 70 | 1323003051 | 276.581 | 0.96 |
| 71 | 1323003052 | 245.826 | 0.93 |
| 72 | 1323003053 | 108.47 | 0.97 |
| 73 | 1323003054 | 111.21 | 0.97 |
| 74 | 1323003055 | 93.7112 | 0.93 |
| 75 | 1323003056 | 166.61 | 0.92 |
| 76 | 1323003563 | 124.067 | 0.94 |

**Supplementary table 3 : Per sample per exon coverage of the DPYD gene in our cohort samples.**

| **Sample ID** | **DPYD_exon1** | **DPYD_exon10** | **DPYD_exon11** | **DPYD_exon12** | **DPYD_exon13** | **DPYD_exon14** | **DPYD_exon15** | **DPYD_exon16** | **DPYD_exon17** | **DPYD_exon18** | **DPYD_exon19** | **DPYD_exon2** | **DPYD_exon20** | **DPYD_exon21** | **DPYD_exon22** | **DPYD_exon23** | **DPYD_exon3** | **DPYD_exon4** | **DPYD_exon5** | **DPYD_exon6** | **DPYD_exon7** | **DPYD_exon8** | **DPYD_exon9** |
| --- | --- | --- | --- | --- | --- | --- | --- | --- | --- | --- | --- | --- | --- | --- | --- | --- | --- | --- | --- | --- | --- | --- | --- |
| 1323002960 | 1 | 1 | 1 | 1 | 1 | 1 | 1 | 1 | 1 | 1 | 1 | 1 | 1 | 1 | 1 | 0.38 | 1 | 1 | 1 | 1 | 1 | 1 | 1 |
| 1323002963 | 1 | 1 | 1 | 1 | 1 | 1 | 1 | 1 | 1 | 1 | 1 | 1 | 1 | 1 | 1 | 0.33 | 1 | 1 | 1 | 1 | 1 | 1 | 1 |
| 1323002965 | 1 | 1 | 1 | 1 | 1 | 1 | 1 | 1 | 1 | 1 | 1 | 1 | 1 | 1 | 1 | 0.57 | 1 | 1 | 1 | 1 | 1 | 1 | 1 |
| 1323002966 | 1 | 1 | 1 | 1 | 1 | 1 | 1 | 1 | 1 | 1 | 1 | 1 | 1 | 1 | 1 | 0.38 | 1 | 1 | 1 | 1 | 1 | 1 | 1 |
| 1323002967 | 1 | 1 | 1 | 1 | 1 | 1 | 1 | 1 | 1 | 1 | 1 | 1 | 1 | 1 | 1 | 0.41 | 1 | 1 | 1 | 1 | 1 | 1 | 1 |
| 1323002968 | 1 | 1 | 1 | 1 | 1 | 1 | 1 | 1 | 1 | 1 | 1 | 1 | 1 | 1 | 1 | 0.61 | 1 | 1 | 1 | 1 | 1 | 1 | 1 |
| 1323002969 | 1 | 1 | 1 | 1 | 1 | 1 | 1 | 1 | 1 | 1 | 1 | 1 | 1 | 1 | 1 | 0.42 | 1 | 1 | 1 | 1 | 1 | 1 | 1 |
| 1323002970 | 1 | 1 | 1 | 1 | 1 | 1 | 1 | 1 | 1 | 1 | 1 | 1 | 1 | 1 | 1 | 0.38 | 1 | 1 | 1 | 1 | 1 | 1 | 1 |
| 1323002971 | 1 | 1 | 1 | 1 | 1 | 1 | 1 | 1 | 1 | 1 | 1 | 1 | 1 | 1 | 1 | 0.38 | 1 | 1 | 1 | 1 | 1 | 1 | 1 |
| 1323002973 | 1 | 1 | 1 | 1 | 1 | 1 | 1 | 1 | 1 | 1 | 1 | 1 | 1 | 1 | 1 | 0.35 | 1 | 1 | 1 | 1 | 1 | 1 | 1 |
| 1323002974 | 1 | 1 | 1 | 1 | 1 | 1 | 1 | 1 | 1 | 1 | 1 | 1 | 1 | 1 | 1 | 0.32 | 1 | 1 | 1 | 1 | 1 | 1 | 1 |
| 1323002975 | 1 | 1 | 1 | 1 | 1 | 1 | 1 | 1 | 1 | 1 | 1 | 1 | 1 | 1 | 1 | 0.34 | 1 | 1 | 1 | 1 | 1 | 1 | 1 |
| 1323002978 | 1 | 1 | 1 | 1 | 1 | 1 | 1 | 1 | 1 | 1 | 1 | 1 | 1 | 1 | 1 | 0.27 | 1 | 1 | 1 | 1 | 1 | 1 | 1 |
| 1323002984 | 1 | 1 | 1 | 1 | 1 | 1 | 1 | 1 | 1 | 1 | 1 | 1 | 1 | 1 | 1 | 0.36 | 1 | 1 | 1 | 1 | 1 | 1 | 1 |
| 1323002987 | 1 | 1 | 1 | 1 | 1 | 1 | 1 | 1 | 1 | 1 | 1 | 1 | 1 | 1 | 1 | 0.4 | 1 | 1 | 1 | 1 | 1 | 1 | 1 |
| 1323002989 | 1 | 1 | 1 | 1 | 1 | 1 | 1 | 1 | 1 | 1 | 1 | 1 | 1 | 1 | 1 | 0.37 | 1 | 1 | 1 | 1 | 1 | 1 | 1 |
| 1323002991 | 1 | 1 | 1 | 1 | 1 | 1 | 1 | 1 | 1 | 1 | 1 | 1 | 1 | 1 | 1 | 0.5 | 1 | 1 | 1 | 1 | 1 | 1 | 1 |
| 1323002992 | 1 | 1 | 1 | 1 | 1 | 1 | 1 | 1 | 1 | 1 | 1 | 1 | 1 | 1 | 1 | 0.34 | 1 | 1 | 1 | 1 | 1 | 1 | 1 |
| 1323002993 | 1 | 1 | 1 | 1 | 1 | 1 | 1 | 1 | 1 | 1 | 1 | 1 | 1 | 1 | 1 | 0.41 | 1 | 1 | 1 | 1 | 1 | 1 | 1 |
| 1323002994 | 1 | 1 | 1 | 1 | 1 | 1 | 1 | 1 | 1 | 1 | 1 | 1 | 1 | 1 | 1 | 0.37 | 1 | 1 | 1 | 1 | 1 | 1 | 1 |
| 1323002996 | 1 | 1 | 1 | 1 | 1 | 1 | 1 | 1 | 1 | 1 | 1 | 1 | 1 | 1 | 1 | 0.33 | 1 | 1 | 1 | 1 | 1 | 1 | 1 |
| 1323002998 | 1 | 1 | 1 | 1 | 1 | 1 | 1 | 1 | 1 | 1 | 1 | 1 | 1 | 1 | 1 | 0.46 | 1 | 1 | 1 | 1 | 1 | 1 | 1 |
| 1323002999 | 1 | 1 | 1 | 1 | 1 | 1 | 1 | 1 | 1 | 1 | 1 | 1 | 1 | 1 | 1 | 0.38 | 1 | 1 | 1 | 1 | 1 | 1 | 1 |
| 1323003000 | 1 | 1 | 1 | 1 | 1 | 1 | 1 | 1 | 1 | 1 | 1 | 1 | 1 | 1 | 1 | 0.39 | 1 | 1 | 1 | 1 | 1 | 1 | 1 |
| 1323003001 | 1 | 1 | 1 | 1 | 1 | 1 | 1 | 1 | 1 | 1 | 1 | 1 | 1 | 1 | 1 | 0.4 | 1 | 1 | 1 | 1 | 1 | 1 | 1 |
| 1323003002 | 1 | 1 | 1 | 1 | 1 | 1 | 1 | 1 | 1 | 1 | 1 | 1 | 1 | 1 | 1 | 0.45 | 1 | 1 | 1 | 1 | 1 | 1 | 1 |
| 1323003003 | 1 | 1 | 1 | 1 | 1 | 1 | 1 | 1 | 1 | 1 | 1 | 1 | 1 | 1 | 1 | 0.39 | 1 | 1 | 1 | 1 | 1 | 1 | 1 |
| 1323003004 | 1 | 1 | 1 | 1 | 1 | 1 | 1 | 1 | 1 | 1 | 1 | 1 | 1 | 1 | 1 | 0.34 | 1 | 1 | 1 | 1 | 1 | 1 | 1 |
| 1323003005 | 1 | 1 | 1 | 1 | 1 | 1 | 1 | 1 | 1 | 1 | 1 | 1 | 1 | 1 | 1 | 0.36 | 1 | 1 | 1 | 1 | 1 | 1 | 1 |
| 1323003006 | 1 | 1 | 1 | 1 | 1 | 1 | 1 | 1 | 1 | 1 | 1 | 1 | 1 | 1 | 1 | 0.44 | 1 | 1 | 1 | 1 | 1 | 1 | 1 |
| 1323003008 | 1 | 1 | 1 | 1 | 1 | 1 | 1 | 1 | 1 | 1 | 1 | 1 | 1 | 1 | 1 | 0.44 | 1 | 1 | 1 | 1 | 1 | 1 | 1 |
| 1323003011 | 1 | 1 | 1 | 1 | 1 | 1 | 1 | 1 | 1 | 1 | 1 | 1 | 1 | 1 | 1 | 0.32 | 1 | 1 | 1 | 1 | 1 | 1 | 1 |
| 1323003012 | 1 | 1 | 1 | 1 | 1 | 1 | 1 | 1 | 1 | 1 | 1 | 1 | 1 | 1 | 1 | 0.38 | 1 | 1 | 1 | 1 | 1 | 1 | 1 |
| 1323003013 | 1 | 1 | 1 | 1 | 1 | 1 | 1 | 1 | 1 | 1 | 1 | 1 | 1 | 1 | 1 | 0.34 | 1 | 1 | 1 | 1 | 1 | 1 | 1 |
| 1323003014 | 1 | 1 | 1 | 1 | 1 | 1 | 1 | 1 | 1 | 1 | 1 | 1 | 1 | 1 | 1 | 0.39 | 1 | 1 | 1 | 1 | 1 | 1 | 1 |
| 1323003015 | 1 | 1 | 1 | 1 | 1 | 1 | 1 | 1 | 1 | 1 | 1 | 1 | 1 | 1 | 1 | 0.39 | 1 | 1 | 1 | 1 | 1 | 1 | 1 |
| 1323003016 | 1 | 1 | 1 | 1 | 1 | 1 | 1 | 1 | 1 | 1 | 1 | 1 | 1 | 1 | 1 | 0.49 | 1 | 1 | 1 | 1 | 1 | 1 | 1 |
| 1323003017 | 1 | 1 | 1 | 1 | 1 | 1 | 1 | 1 | 1 | 1 | 1 | 1 | 1 | 1 | 1 | 0.3 | 1 | 1 | 1 | 1 | 1 | 1 | 1 |
| 1323003018 | 1 | 1 | 1 | 1 | 1 | 1 | 1 | 1 | 1 | 1 | 1 | 1 | 1 | 1 | 1 | 0.37 | 1 | 1 | 1 | 1 | 1 | 1 | 1 |
| 1323003019 | 1 | 1 | 1 | 1 | 1 | 1 | 1 | 1 | 1 | 1 | 1 | 1 | 1 | 1 | 1 | 0.41 | 1 | 1 | 1 | 1 | 1 | 1 | 1 |
| 1323003020 | 1 | 1 | 1 | 1 | 1 | 1 | 1 | 1 | 1 | 1 | 1 | 1 | 1 | 1 | 1 | 0.33 | 1 | 1 | 1 | 1 | 1 | 1 | 1 |
| 1323003022 | 1 | 1 | 1 | 1 | 1 | 1 | 1 | 1 | 1 | 1 | 1 | 1 | 1 | 1 | 1 | 0.31 | 1 | 1 | 1 | 1 | 1 | 1 | 1 |
| 1323003023 | 1 | 1 | 1 | 1 | 1 | 1 | 1 | 1 | 1 | 1 | 1 | 1 | 1 | 1 | 1 | 0.29 | 1 | 1 | 1 | 1 | 1 | 1 | 1 |
| 1323003024 | 1 | 1 | 1 | 1 | 1 | 1 | 1 | 1 | 1 | 1 | 1 | 1 | 1 | 1 | 1 | 0.33 | 1 | 1 | 1 | 1 | 1 | 1 | 1 |
| 1323003025 | 1 | 1 | 1 | 1 | 1 | 1 | 1 | 1 | 1 | 1 | 1 | 1 | 1 | 1 | 1 | 0.41 | 1 | 1 | 1 | 1 | 1 | 1 | 1 |
| 1323003026 | 1 | 1 | 1 | 1 | 1 | 1 | 1 | 1 | 1 | 1 | 1 | 1 | 1 | 1 | 1 | 0.35 | 1 | 1 | 1 | 1 | 1 | 1 | 1 |
| 1323003027 | 1 | 1 | 1 | 1 | 1 | 1 | 1 | 1 | 1 | 1 | 1 | 1 | 1 | 1 | 1 | 0.38 | 1 | 1 | 1 | 1 | 1 | 1 | 1 |
| 1323003028 | 1 | 1 | 1 | 1 | 1 | 1 | 1 | 1 | 1 | 1 | 1 | 1 | 1 | 1 | 1 | 0.34 | 1 | 1 | 1 | 1 | 1 | 1 | 1 |
| 1323003029 | 1 | 1 | 1 | 1 | 1 | 1 | 1 | 1 | 1 | 1 | 1 | 1 | 1 | 1 | 1 | 0.2 | 1 | 1 | 1 | 1 | 1 | 1 | 1 |
| 1323003030 | 1 | 1 | 1 | 1 | 1 | 1 | 1 | 1 | 1 | 1 | 1 | 1 | 1 | 1 | 1 | 0.23 | 1 | 1 | 1 | 1 | 1 | 1 | 1 |
| 1323003031 | 0.75 | 1 | 1 | 1 | 1 | 1 | 1 | 1 | 1 | 1 | 1 | 1 | 1 | 1 | 1 | 0.16 | 1 | 1 | 1 | 1 | 1 | 1 | 1 |
| 1323003032 | 1 | 1 | 1 | 1 | 1 | 1 | 1 | 1 | 1 | 1 | 1 | 1 | 1 | 1 | 1 | 0.25 | 1 | 1 | 1 | 1 | 1 | 1 | 1 |
| 1323003033 | 1 | 1 | 1 | 1 | 1 | 1 | 1 | 1 | 1 | 1 | 1 | 1 | 1 | 1 | 1 | 0.22 | 1 | 1 | 1 | 1 | 1 | 1 | 1 |
| 1323003034 | 1 | 1 | 1 | 1 | 1 | 1 | 1 | 1 | 1 | 1 | 1 | 1 | 1 | 1 | 1 | 0.35 | 1 | 1 | 1 | 1 | 1 | 1 | 1 |
| 1323003035 | 1 | 1 | 1 | 1 | 1 | 1 | 1 | 1 | 1 | 1 | 1 | 1 | 1 | 1 | 1 | 0.26 | 1 | 1 | 1 | 1 | 1 | 1 | 1 |
| 1323003036 | 1 | 1 | 1 | 1 | 1 | 1 | 1 | 1 | 1 | 1 | 1 | 1 | 1 | 1 | 1 | 0.35 | 1 | 1 | 1 | 1 | 1 | 1 | 1 |
| 1323003037 | 1 | 1 | 1 | 1 | 1 | 1 | 1 | 1 | 1 | 1 | 1 | 1 | 1 | 1 | 1 | 0.26 | 1 | 1 | 1 | 1 | 1 | 1 | 1 |
| 1323003038 | 1 | 1 | 1 | 1 | 1 | 1 | 1 | 1 | 1 | 1 | 1 | 1 | 1 | 1 | 1 | 0.28 | 1 | 1 | 1 | 1 | 1 | 1 | 1 |
| 1323003039 | 1 | 1 | 1 | 1 | 1 | 1 | 1 | 1 | 1 | 1 | 1 | 1 | 1 | 1 | 1 | 0.25 | 1 | 1 | 1 | 1 | 1 | 1 | 1 |
| 1323003040 | 1 | 1 | 1 | 1 | 1 | 1 | 1 | 1 | 1 | 1 | 1 | 1 | 1 | 1 | 1 | 0.34 | 1 | 1 | 1 | 1 | 1 | 1 | 1 |
| 1323003041 | 1 | 1 | 1 | 1 | 1 | 1 | 1 | 1 | 1 | 1 | 1 | 1 | 1 | 1 | 1 | 0.28 | 1 | 1 | 1 | 1 | 1 | 1 | 1 |
| 1323003042 | 1 | 1 | 1 | 1 | 1 | 1 | 1 | 1 | 1 | 1 | 1 | 1 | 1 | 1 | 1 | 0.29 | 1 | 1 | 1 | 1 | 1 | 1 | 1 |
| 1323003043 | 1 | 1 | 1 | 1 | 1 | 1 | 1 | 1 | 1 | 1 | 1 | 1 | 1 | 1 | 1 | 0.18 | 1 | 1 | 1 | 1 | 1 | 1 | 1 |
| 1323003044 | 1 | 1 | 1 | 1 | 1 | 1 | 1 | 1 | 1 | 1 | 1 | 1 | 1 | 1 | 1 | 0.25 | 1 | 1 | 1 | 1 | 1 | 1 | 1 |
| 1323003046 | 1 | 1 | 1 | 1 | 1 | 1 | 1 | 1 | 1 | 1 | 1 | 1 | 1 | 1 | 1 | 0.28 | 1 | 1 | 1 | 1 | 1 | 1 | 1 |
| 1323003047 | 1 | 1 | 1 | 1 | 1 | 1 | 1 | 1 | 1 | 1 | 1 | 1 | 1 | 1 | 1 | 0.46 | 1 | 1 | 1 | 1 | 1 | 1 | 1 |
| 1323003048 | 1 | 1 | 1 | 1 | 1 | 1 | 1 | 1 | 1 | 1 | 1 | 1 | 1 | 1 | 1 | 0.28 | 1 | 1 | 1 | 1 | 1 | 1 | 1 |
| 1323003049 | 1 | 1 | 1 | 1 | 1 | 1 | 1 | 1 | 1 | 1 | 1 | 1 | 1 | 1 | 1 | 0.22 | 1 | 1 | 1 | 1 | 1 | 1 | 1 |
| 1323003050 | 0.95 | 1 | 1 | 1 | 1 | 1 | 1 | 1 | 1 | 1 | 1 | 1 | 1 | 1 | 1 | 0.34 | 1 | 1 | 1 | 1 | 1 | 1 | 1 |
| 1323003051 | 1 | 1 | 1 | 1 | 1 | 1 | 1 | 1 | 1 | 1 | 1 | 1 | 1 | 1 | 1 | 0.32 | 1 | 1 | 1 | 1 | 1 | 1 | 1 |
| 1323003052 | 1 | 1 | 1 | 1 | 1 | 1 | 1 | 1 | 1 | 1 | 1 | 1 | 1 | 1 | 1 | 0.41 | 1 | 1 | 1 | 1 | 1 | 1 | 1 |
| 1323003053 | 1 | 1 | 1 | 1 | 1 | 1 | 1 | 1 | 1 | 1 | 1 | 1 | 1 | 1 | 1 | 0.22 | 1 | 1 | 1 | 1 | 1 | 1 | 1 |
| 1323003054 | 1 | 1 | 1 | 1 | 1 | 1 | 1 | 1 | 1 | 1 | 1 | 1 | 1 | 1 | 1 | 0.24 | 1 | 1 | 1 | 1 | 1 | 1 | 1 |
| 1323003055 | 1 | 1 | 1 | 1 | 1 | 1 | 1 | 1 | 1 | 1 | 1 | 1 | 1 | 1 | 1 | 0.33 | 1 | 1 | 1 | 1 | 1 | 1 | 1 |
| 1323003056 | 1 | 1 | 1 | 1 | 1 | 1 | 1 | 1 | 1 | 1 | 1 | 1 | 1 | 1 | 1 | 0.43 | 1 | 1 | 1 | 1 | 1 | 1 | 1 |
| 1323003563 | 1 | 1 | 1 | 1 | 1 | 1 | 1 | 1 | 1 | 1 | 1 | 1 | 1 | 1 | 1 | 0.32 | 1 | 1 | 1 | 1 | 1 | 1 | 1 |

**Supplementary table 4 : Per sample per exon depth of the DPYD gene in our cohort samples.**

| **Sample ID** | **DPYD_exon1** | **DPYD_exon10** | **DPYD_exon11** | **DPYD_exon12** | **DPYD_exon13** | **DPYD_exon14** | **DPYD_exon15** | **DPYD_exon16** | **DPYD_exon17** | **DPYD_exon18** | **DPYD_exon19** | **DPYD_exon2** | **DPYD_exon20** | **DPYD_exon21** | **DPYD_exon22** | **DPYD_exon23** | **DPYD_exon3** | **DPYD_exon4** | **DPYD_exon5** | **DPYD_exon6** | **DPYD_exon7** | **DPYD_exon8** | **DPYD_exon9** |
| --- | --- | --- | --- | --- | --- | --- | --- | --- | --- | --- | --- | --- | --- | --- | --- | --- | --- | --- | --- | --- | --- | --- | --- |
| 1323002960 | 117.62 | 142.96 | 150.85 | 164.16 | 160.07 | 147.65 | 107.25 | 110.8 | 207.48 | 133.98 | 136.29 | 104.64 | 113.43 | 132.11 | 103.16 | 139.96 | 88.71 | 108.67 | 120.56 | 149.59 | 117.76 | 144.32 | 90.21 |
| 1323002963 | 98.72 | 156.47 | 117.22 | 128.06 | 145.9 | 105.45 | 67.64 | 79.98 | 172.23 | 107.58 | 99.78 | 105.73 | 86.28 | 98.83 | 88.94 | 111.44 | 84.35 | 52.36 | 105.72 | 121.53 | 121.73 | 100.06 | 59.31 |
| 1323002965 | 85.51 | 106.16 | 126.23 | 150.35 | 141.45 | 102.82 | 74.78 | 89.75 | 139.86 | 118.12 | 122.64 | 84.08 | 123.91 | 103.84 | 111.67 | 113.99 | 58.71 | 57.63 | 112.87 | 135.93 | 106.84 | 79.13 | 86.96 |
| 1323002966 | 136.44 | 175 | 133.57 | 183.74 | 181.05 | 134.54 | 105.16 | 101.21 | 266.43 | 141.86 | 145.2 | 94.9 | 131.24 | 144.12 | 156.18 | 168.02 | 127.27 | 90.34 | 142.4 | 158.59 | 144.67 | 124.38 | 109.44 |
| 1323002967 | 150.18 | 187.17 | 146.46 | 186.5 | 211.47 | 173.77 | 129.25 | 135.48 | 212.52 | 156.35 | 153.69 | 126.21 | 156.28 | 162.98 | 138.34 | 184.01 | 69.78 | 80.74 | 177.27 | 182.06 | 167.6 | 132.68 | 112.05 |
| 1323002968 | 120.03 | 183.98 | 147.47 | 164.94 | 195.19 | 142.41 | 87.48 | 117.6 | 203.77 | 139.13 | 145.41 | 118.03 | 130.81 | 126.82 | 136.75 | 144.37 | 82.05 | 72.88 | 128.56 | 160 | 150.67 | 113.69 | 100.84 |
| 1323002969 | 184.87 | 238.94 | 182.54 | 265.46 | 271.86 | 170.22 | 186.43 | 215.1 | 324.08 | 219.13 | 189.36 | 172.67 | 225.77 | 238.53 | 203.9 | 235.53 | 136.81 | 94.39 | 232.43 | 279.72 | 206.46 | 185.97 | 179.67 |
| 1323002970 | 255.49 | 282.45 | 235.51 | 334.32 | 307.75 | 207.01 | 165.19 | 172.26 | 369.46 | 233.77 | 262.71 | 199.5 | 229.7 | 249.32 | 207.82 | 242.6 | 181.34 | 157.19 | 271.72 | 298.28 | 203.59 | 183.7 | 145.32 |
| 1323002971 | 346.13 | 447.85 | 304.91 | 470.98 | 473.56 | 338.07 | 251.52 | 338.89 | 535.49 | 314.84 | 358.91 | 256.88 | 334.22 | 349.09 | 316.65 | 429.12 | 227.64 | 223.02 | 339.97 | 475.06 | 368.32 | 276.32 | 246.02 |
| 1323002973 | 86.38 | 123.24 | 81.07 | 121.05 | 133.83 | 81.03 | 73.71 | 96.87 | 163.69 | 107.39 | 104.88 | 80.79 | 88.56 | 102.22 | 93.77 | 123.61 | 77.43 | 58.25 | 97.76 | 118.44 | 76.01 | 110.83 | 83.66 |
| 1323002974 | 59.97 | 79.71 | 60.7 | 88.46 | 91.77 | 60.42 | 44.28 | 42.06 | 100.54 | 61.83 | 60.74 | 57.43 | 62.22 | 83.17 | 64.57 | 70.59 | 38.01 | 46.52 | 66.72 | 81.38 | 73.43 | 57.07 | 35.97 |
| 1323002975 | 134.79 | 120.12 | 88 | 108.92 | 108.43 | 87.25 | 59.68 | 77.74 | 145.4 | 102.61 | 82.14 | 97.26 | 96.84 | 102.03 | 86.6 | 119.92 | 71.58 | 55.17 | 84.44 | 110.48 | 88.27 | 72.52 | 78.14 |
| 1323002978 | 186 | 162.96 | 120.64 | 189.41 | 176.19 | 127.76 | 112.65 | 114.48 | 229.01 | 157.23 | 159.53 | 105.42 | 151.92 | 123.72 | 101.38 | 170.18 | 109.42 | 113.7 | 131.98 | 158.97 | 118.88 | 110.52 | 110.89 |
| 1323002984 | 210.33 | 219.83 | 146.1 | 196.69 | 180.42 | 134.67 | 97.51 | 121.64 | 257.65 | 174.73 | 141.66 | 114.86 | 141.15 | 164.4 | 158.17 | 168.82 | 124.01 | 69.16 | 168.69 | 181.78 | 129.49 | 172.81 | 106.76 |
| 1323002987 | 104.49 | 167.86 | 109.84 | 139.45 | 145.83 | 121.9 | 91.88 | 88.2 | 153.98 | 114.09 | 111.73 | 81.22 | 115.36 | 92.9 | 94.43 | 111.87 | 66.33 | 47.51 | 119.94 | 159.04 | 117.78 | 102.08 | 77.46 |
| 1323002989 | 161.79 | 201.59 | 121.95 | 176.58 | 191.16 | 124.72 | 89.93 | 132.75 | 236.21 | 119.97 | 168.83 | 110.72 | 172.02 | 145.15 | 136.19 | 182.7 | 103.6 | 85.94 | 152.19 | 146.85 | 139.17 | 130.41 | 127.96 |
| 1323002991 | 214.23 | 213.91 | 208.81 | 229.43 | 231.55 | 136.62 | 157.07 | 162.36 | 284.7 | 173.17 | 172.76 | 127.42 | 199.59 | 180.97 | 179.03 | 212.01 | 121.35 | 108.42 | 230.77 | 230.46 | 197.44 | 211.3 | 144.84 |
| 1323002992 | 111.82 | 104.76 | 80.76 | 98.65 | 99.52 | 63.98 | 58.28 | 75.71 | 104.12 | 67.82 | 87.41 | 49.4 | 75.07 | 71.35 | 60.05 | 66.34 | 35.52 | 45.82 | 88.85 | 103.91 | 75.24 | 54.48 | 55.97 |
| 1323002993 | 125.87 | 183.36 | 170.55 | 194.09 | 208.93 | 142.87 | 116.04 | 138.04 | 252.69 | 131.1 | 161.22 | 128.28 | 157.85 | 157.32 | 157.77 | 154.42 | 111.01 | 76.69 | 164.34 | 196.33 | 136.68 | 135.06 | 84.32 |
| 1323002994 | 191.38 | 226.88 | 178.9 | 248.95 | 251.9 | 147.42 | 130.96 | 136.96 | 270.99 | 195.38 | 210.1 | 139.35 | 159.91 | 182.83 | 184.57 | 232.51 | 109.71 | 102.25 | 205.17 | 198.77 | 192.12 | 165.09 | 132.64 |
| 1323002996 | 104.87 | 131.06 | 130.24 | 128.37 | 126.4 | 138.99 | 84.09 | 90.93 | 150.81 | 84.13 | 109.92 | 63.6 | 113.78 | 120.17 | 86.46 | 125.5 | 56.47 | 64.26 | 121.12 | 126.51 | 109.17 | 116.01 | 101.47 |
| 1323002998 | 371.97 | 407.88 | 306.78 | 418.88 | 467.36 | 302.3 | 217.81 | 273.42 | 507.04 | 351.76 | 305.01 | 261.14 | 320.38 | 321.84 | 294.52 | 372.63 | 234.84 | 153.03 | 363.36 | 404.59 | 326.74 | 265.76 | 220.49 |
| 1323002999 | 86.62 | 78.2 | 58.05 | 74.72 | 88.5 | 36.36 | 46.41 | 69.25 | 72.6 | 52.31 | 67.92 | 44.33 | 52.27 | 69.85 | 60.62 | 70.06 | 39.93 | 18.59 | 85.9 | 51.33 | 62.1 | 46.63 | 49.07 |
| 1323003000 | 71.23 | 91.39 | 90.73 | 111.21 | 115.61 | 75.72 | 59.91 | 61.85 | 123.08 | 80.18 | 92.15 | 75.93 | 69.47 | 76.83 | 71.57 | 83.31 | 47.82 | 40 | 90.8 | 80.74 | 82.66 | 75.78 | 68.97 |
| 1323003001 | 181.05 | 129.74 | 144.4 | 181.56 | 229.53 | 139.68 | 138.96 | 108.62 | 241.79 | 108.28 | 133.89 | 114.11 | 146.21 | 124.83 | 115.65 | 152.84 | 132.06 | 62.81 | 141.07 | 169.96 | 107.32 | 154.98 | 108.12 |
| 1323003002 | 91.67 | 98.8 | 100.64 | 131.15 | 132.33 | 91.04 | 77.2 | 71.44 | 125.14 | 95.47 | 96.24 | 69.13 | 91.44 | 106.15 | 89.2 | 90.18 | 57.76 | 71.63 | 119.84 | 113.4 | 83.5 | 87.15 | 66.71 |
| 1323003003 | 141.15 | 175.98 | 136.55 | 166.98 | 178.79 | 141.42 | 109.62 | 114.04 | 218.95 | 142.89 | 133.9 | 124.28 | 140.76 | 125.77 | 125.6 | 174.12 | 65.28 | 97.65 | 162.09 | 176.26 | 113.22 | 132.7 | 79.47 |
| 1323003004 | 108.79 | 138.77 | 107.14 | 149.77 | 142.16 | 92.67 | 80.1 | 90.95 | 184.93 | 112.81 | 109.46 | 79.41 | 94.56 | 108.41 | 94.14 | 125.1 | 97.64 | 78.22 | 127.31 | 162.56 | 105.76 | 107.88 | 48.4 |
| 1323003005 | 108.56 | 98.76 | 87.73 | 121.37 | 162.28 | 96.02 | 64.43 | 105.76 | 153.86 | 91.9 | 120.8 | 74.23 | 113.72 | 99.82 | 104.78 | 115.11 | 46.01 | 64.83 | 128.13 | 115.8 | 84.99 | 107.39 | 87.47 |
| 1323003006 | 241.33 | 222.84 | 213.34 | 247.48 | 285.06 | 197.52 | 198.29 | 155.89 | 377.8 | 199.69 | 245.52 | 158.28 | 202.16 | 210.55 | 209.85 | 254.06 | 177.81 | 117.99 | 229.48 | 241.54 | 212.13 | 199.75 | 156.15 |
| 1323003008 | 96.23 | 110.95 | 91.06 | 110.4 | 120.32 | 81.67 | 51.04 | 64.33 | 137.58 | 82.75 | 74.03 | 61.06 | 80.15 | 109.51 | 104.21 | 96.6 | 48.24 | 40.33 | 77.52 | 122.16 | 112.11 | 95.51 | 43.74 |
| 1323003011 | 129.79 | 139.49 | 117.43 | 148.41 | 169.63 | 120.36 | 112.2 | 103.99 | 221.85 | 132.39 | 112.19 | 103.32 | 133.78 | 152.78 | 100.41 | 138.46 | 88.39 | 65.1 | 143.44 | 139.44 | 141.8 | 133.06 | 89.19 |
| 1323003012 | 135.28 | 206.21 | 168.08 | 196.36 | 241.03 | 139.54 | 108.59 | 140.77 | 239.06 | 147.72 | 168.45 | 150 | 189.42 | 151.13 | 170.48 | 199.99 | 112.96 | 102.47 | 179.6 | 190.79 | 162.28 | 157.43 | 111.23 |
| 1323003013 | 133.95 | 151.54 | 101.41 | 168.21 | 178.38 | 104.93 | 103.46 | 87.68 | 204.63 | 119.17 | 121.92 | 80.2 | 123.08 | 104.06 | 115.35 | 124.91 | 71.84 | 67.56 | 125.98 | 140.67 | 134.15 | 142.53 | 72.62 |
| 1323003014 | 136.08 | 125.46 | 113.36 | 164.01 | 205.66 | 126.22 | 108.39 | 121.83 | 247.73 | 118.32 | 148.55 | 104.91 | 158.06 | 157.99 | 164.71 | 169.36 | 70.8 | 73.23 | 155.4 | 205.53 | 142.74 | 142.67 | 98.94 |
| 1323003015 | 128.18 | 159.68 | 118.72 | 146.21 | 144.97 | 119.46 | 67.06 | 134.12 | 166.28 | 112.23 | 137.43 | 110.62 | 125.81 | 136.63 | 131.38 | 153.02 | 71.06 | 81.98 | 117.96 | 148.29 | 117.76 | 115.49 | 113.64 |
| 1323003016 | 236.38 | 219.21 | 203.15 | 266.23 | 274.79 | 199.19 | 132.55 | 197.23 | 329.99 | 194.22 | 223.13 | 190.95 | 165.98 | 218.47 | 227.73 | 233.98 | 153.73 | 108.2 | 207.59 | 271.97 | 177.59 | 207.4 | 126.92 |
| 1323003017 | 212.41 | 244.21 | 189.22 | 235.05 | 244.73 | 179.08 | 177.9 | 141.42 | 307.69 | 168.54 | 173.28 | 180.33 | 195.12 | 175.85 | 178.67 | 200.07 | 119.65 | 120.86 | 174.97 | 262.15 | 152.59 | 205.89 | 141.58 |
| 1323003018 | 290.49 | 323.39 | 222.85 | 294.19 | 300.4 | 239.79 | 235.78 | 256.11 | 390.29 | 226.98 | 250.07 | 206.4 | 238.55 | 267.27 | 241.96 | 282.94 | 183.27 | 116.7 | 257.02 | 308.46 | 235.62 | 226.01 | 206.93 |
| 1323003019 | 283.38 | 364.35 | 253.34 | 326.35 | 402.37 | 258.66 | 249.91 | 225.49 | 457.03 | 303.38 | 325.73 | 256.45 | 259.03 | 310.11 | 211.82 | 263.67 | 179.86 | 151.3 | 280.97 | 322.97 | 252.13 | 230.16 | 225.94 |
| 1323003020 | 157.36 | 151.61 | 106.64 | 183.36 | 181.3 | 121.9 | 87.32 | 89.63 | 221.04 | 149.02 | 156.13 | 134.36 | 139.11 | 151.83 | 113.94 | 149.13 | 92.8 | 72.57 | 104.23 | 150.4 | 108.16 | 131.36 | 96.69 |
| 1323003022 | 214.85 | 199.65 | 187.31 | 209.71 | 248.75 | 170.51 | 133.64 | 149.96 | 281.39 | 209.08 | 202.32 | 144.02 | 182.63 | 168.58 | 171.82 | 236.56 | 124.88 | 81.01 | 203.25 | 245.7 | 186.01 | 203.08 | 140.03 |
| 1323003023 | 181.15 | 177.53 | 184.09 | 151.06 | 210.9 | 143.16 | 114.07 | 149.51 | 248.05 | 160.82 | 172.44 | 79.58 | 121.86 | 171.05 | 135.98 | 150.84 | 103.92 | 96.14 | 152.84 | 169.46 | 139.88 | 154.58 | 115.69 |
| 1323003024 | 143.13 | 213.26 | 143.46 | 194.88 | 193.43 | 146.79 | 133.42 | 146.61 | 266.07 | 152.51 | 153.45 | 145.5 | 176.43 | 127.91 | 129.69 | 205.37 | 126.67 | 114.56 | 176.68 | 197.88 | 124.38 | 167.92 | 110.31 |
| 1323003025 | 217.38 | 211.31 | 158.33 | 214.43 | 267.49 | 146.21 | 133.62 | 167.1 | 296.35 | 182.06 | 182.91 | 154.19 | 205.92 | 147.09 | 199.19 | 216.99 | 135.64 | 111.67 | 180.29 | 243.79 | 162.41 | 185.83 | 121 |
| 1323003026 | 148.44 | 242.76 | 194.5 | 229.76 | 222.6 | 192.01 | 143 | 186.85 | 263.23 | 215.26 | 146.62 | 161.87 | 177.29 | 167.62 | 175.52 | 209.89 | 115.58 | 123.1 | 178 | 262.89 | 175.83 | 157.17 | 130.87 |
| 1323003027 | 113.15 | 111.58 | 92.98 | 123.46 | 91.98 | 91.98 | 65.86 | 81.7 | 139.14 | 124.99 | 73.11 | 61.63 | 91.21 | 62.37 | 88.91 | 83.64 | 64.2 | 67.48 | 101.46 | 114.63 | 78.77 | 79.68 | 64.45 |
| 1323003028 | 99.59 | 76.86 | 67.82 | 80.16 | 64.79 | 51.6 | 38.94 | 62.37 | 95.52 | 66.77 | 80.87 | 67.11 | 65.22 | 54.89 | 45.77 | 75.24 | 54.33 | 36.13 | 76.77 | 79.38 | 77.16 | 54.38 | 44.61 |
| 1323003029 | 73.51 | 55.22 | 29.24 | 47.16 | 67.2 | 46.6 | 28.8 | 40.05 | 78.73 | 48.65 | 53.8 | 33.18 | 38.87 | 40.44 | 53.48 | 59.65 | 24.18 | 24.48 | 51.49 | 73.03 | 40.4 | 44.09 | 52.31 |
| 1323003030 | 174.74 | 121.54 | 115.16 | 154.05 | 145.29 | 97.1 | 71.88 | 145.7 | 181.97 | 127.67 | 133.83 | 106.11 | 112.07 | 111.21 | 91.55 | 122.93 | 106.07 | 94.59 | 117.93 | 126.86 | 110.41 | 116.65 | 78.67 |
| 1323003031 | 100.21 | 58.04 | 55.66 | 58.85 | 60.74 | 59.79 | 44.74 | 44.88 | 112.55 | 55.42 | 52.84 | 39.58 | 41.15 | 49.31 | 52.41 | 54.42 | 46.33 | 45.99 | 41.14 | 81.26 | 45.76 | 46.35 | 56.21 |
| 1323003032 | 85.33 | 71.92 | 53.8 | 72.43 | 61.58 | 30.27 | 32.28 | 56.79 | 74.87 | 57.07 | 51.78 | 44.27 | 49.31 | 52.97 | 27.11 | 71.73 | 37.23 | 31.35 | 50.28 | 67.01 | 69.65 | 66.47 | 35.72 |
| 1323003033 | 123.85 | 112.9 | 72.61 | 98.89 | 101.81 | 77.76 | 69.59 | 77.3 | 141.29 | 80.17 | 83.67 | 63.65 | 87.91 | 60.33 | 82.28 | 86.83 | 83.34 | 51.13 | 88.8 | 92.85 | 68.4 | 67.82 | 70.19 |
| 1323003034 | 118.21 | 107.37 | 83.15 | 117.58 | 114.09 | 107 | 46.83 | 58.65 | 121.15 | 116.73 | 114.13 | 75.22 | 64.74 | 101.15 | 84.09 | 116.2 | 64.04 | 29.43 | 98.62 | 117.27 | 90.27 | 77.35 | 66.81 |
| 1323003035 | 99.15 | 102.56 | 54.72 | 71.06 | 73.91 | 63.13 | 56.13 | 43.83 | 98.03 | 82.63 | 64.92 | 62.46 | 64.48 | 71.81 | 65.96 | 64.95 | 54.81 | 28.84 | 72.48 | 81.36 | 68.54 | 53.35 | 38.07 |
| 1323003036 | 82.97 | 109.26 | 70.04 | 75.4 | 73.95 | 72.48 | 59.51 | 61.48 | 118.91 | 76.14 | 77.94 | 62.91 | 46.26 | 99.33 | 64.6 | 61.49 | 77.27 | 43.9 | 83.16 | 81.72 | 75.96 | 47.18 | 43.81 |
| 1323003037 | 69.74 | 68.35 | 46.25 | 53.71 | 78.47 | 41.01 | 37.2 | 52.79 | 79.77 | 65.73 | 52.8 | 32.51 | 49.74 | 53.17 | 44.07 | 57.61 | 33.8 | 33.69 | 51.38 | 72.02 | 47 | 44.5 | 31.26 |
| 1323003038 | 136.21 | 119.37 | 101.09 | 130.76 | 157.38 | 86.27 | 95.19 | 87.61 | 158.06 | 112.53 | 110.28 | 86.99 | 95.64 | 107.9 | 97.84 | 111.67 | 81.48 | 75.44 | 111.94 | 139.03 | 81.87 | 84.99 | 74.18 |
| 1323003039 | 60.51 | 91.86 | 79.27 | 100.11 | 92.92 | 77.73 | 51.96 | 67.92 | 129.17 | 67.1 | 83.32 | 79.21 | 92.83 | 61.01 | 69.98 | 89.3 | 66.96 | 54.17 | 92.25 | 90.07 | 63.54 | 75.4 | 52.81 |
| 1323003040 | 82.08 | 109.78 | 93.18 | 112.86 | 116.75 | 62.83 | 82.59 | 74.6 | 119.55 | 80.41 | 100.47 | 83.29 | 77.43 | 82.31 | 80.31 | 81.02 | 65.23 | 49.57 | 106.85 | 95.24 | 80.54 | 71.16 | 70.78 |
| 1323003041 | 84.74 | 68.87 | 48.5 | 62.83 | 95.26 | 43.26 | 31.39 | 55.38 | 72.02 | 57.07 | 58.29 | 43.95 | 61.25 | 62.94 | 45.43 | 52.76 | 40.87 | 26.34 | 56.67 | 76.57 | 36.67 | 75.68 | 50.39 |
| 1323003042 | 119.46 | 119.01 | 73.13 | 114.94 | 129.11 | 81.49 | 56.19 | 75.98 | 116.12 | 90.84 | 82.62 | 65.82 | 64.19 | 105.19 | 93.78 | 91.89 | 57.77 | 44.52 | 83.67 | 91.36 | 94.09 | 92.19 | 67.87 |
| 1323003043 | 83.05 | 55.95 | 40.27 | 52.41 | 52.28 | 32.9 | 30.88 | 65.61 | 113.82 | 57.37 | 58.53 | 37.93 | 36.12 | 45.83 | 32.33 | 64.22 | 40.27 | 20.41 | 49.2 | 72.08 | 55.84 | 36.5 | 35.1 |
| 1323003044 | 67.56 | 62.31 | 45.92 | 58.96 | 64.96 | 39.65 | 30.3 | 45.21 | 77.47 | 41.62 | 60.5 | 60.27 | 65.93 | 38.53 | 54.57 | 56.84 | 51.55 | 24.34 | 50.97 | 74.09 | 49.9 | 64.83 | 26.73 |
| 1323003046 | 61.59 | 72.28 | 60.45 | 101.85 | 100.89 | 76.35 | 87.46 | 50.46 | 130.51 | 74.19 | 94.9 | 64.46 | 56.09 | 85.81 | 67.23 | 75.42 | 57.7 | 38.28 | 76.81 | 81.15 | 105.57 | 74.66 | 47.18 |
| 1323003047 | 89.79 | 96.24 | 80.05 | 84.76 | 87.5 | 65.25 | 51.35 | 44.89 | 110.27 | 77.44 | 77.99 | 63.11 | 76.24 | 62.86 | 78.49 | 90.49 | 58.9 | 51.43 | 80.08 | 87.35 | 68.05 | 58.9 | 67.19 |
| 1323003048 | 67.51 | 81.56 | 44.29 | 66.08 | 64.77 | 62.64 | 44.43 | 38.25 | 79.6 | 62.92 | 58.65 | 57.25 | 52.58 | 41.9 | 63.62 | 64.75 | 50.65 | 17.27 | 54.71 | 57.19 | 50.9 | 44.65 | 26.94 |
| 1323003049 | 94.18 | 73.39 | 73.54 | 89.65 | 108.56 | 71.41 | 92.19 | 73.55 | 106.39 | 91.39 | 70 | 68.68 | 69.04 | 77.3 | 71.32 | 89.85 | 73.07 | 50.58 | 66.14 | 96.65 | 65.67 | 64.76 | 84.7 |
| 1323003050 | 80.38 | 60.31 | 32.36 | 59.55 | 68.81 | 47.93 | 29.48 | 48.04 | 82.92 | 57.01 | 54.76 | 40.56 | 55.81 | 48.09 | 32.77 | 51.69 | 37.4 | 18.52 | 42.57 | 63.66 | 35.82 | 46.18 | 31.99 |
| 1323003051 | 213.28 | 186.91 | 168.33 | 225.77 | 208.63 | 194.93 | 137.36 | 140.5 | 255.68 | 191.98 | 172.5 | 142.6 | 169.67 | 151.26 | 131.91 | 208.42 | 126.46 | 112.25 | 159.21 | 161.59 | 156.79 | 138.05 | 101.09 |
| 1323003052 | 147.31 | 187.24 | 147.35 | 207.75 | 200.07 | 141.33 | 115.07 | 104.11 | 254.08 | 139.25 | 174.22 | 150.86 | 154.53 | 162.09 | 130.45 | 172.99 | 129.52 | 71.47 | 162.27 | 179.41 | 126.84 | 149.59 | 87.07 |
| 1323003053 | 75.46 | 56.3 | 56.7 | 59.89 | 76.47 | 44.84 | 52.91 | 58.86 | 93.33 | 74.14 | 64.9 | 58.58 | 47.19 | 58.38 | 55.18 | 67.8 | 44.57 | 30.61 | 64.9 | 71.37 | 59.72 | 48.5 | 32.19 |
| 1323003054 | 72.36 | 78.99 | 61.22 | 88.95 | 74.37 | 66.66 | 53.1 | 63.06 | 140.16 | 63.88 | 76.22 | 49.22 | 42.59 | 65.94 | 58.65 | 72.18 | 40.87 | 21.43 | 70.65 | 62.27 | 58.23 | 44.7 | 35.35 |
| 1323003055 | 62.46 | 50.94 | 51.91 | 75.81 | 86.13 | 67.64 | 41.55 | 42.32 | 84.77 | 49.63 | 61.59 | 52.53 | 74.31 | 55.16 | 48.26 | 57.94 | 41.59 | 40.88 | 66.2 | 93.45 | 38.04 | 58.11 | 33.57 |
| 1323003056 | 123.59 | 115.21 | 96.09 | 120.34 | 160.88 | 115.21 | 62.46 | 93.24 | 155.96 | 73.1 | 98.68 | 74.43 | 83.19 | 82.98 | 86.13 | 109.95 | 86.77 | 67.59 | 109.22 | 115.55 | 64.68 | 87.81 | 70.89 |
| 1323003563 | 114.28 | 77.96 | 76.05 | 111.57 | 110.17 | 54.95 | 73.97 | 65.98 | 133.18 | 77.88 | 97.3 | 65.45 | 74.97 | 58.9 | 81.75 | 67.81 | 56.78 | 40.61 | 75.22 | 99.9 | 97.59 | 50.4 | 55.09 |

**Supplementary table 5 : List of multi allelic variant identified in our cohort**

| **#CHROM** | **POS** | **ID** | **REF** | **ALT** | **QUAL** | **FILTER** | **INFO** |
| --- | --- | --- | --- | --- | --- | --- | --- |
| chr1 | 97158472 | . | GACACACACAC | GACACACACACACAC | 30.95 | PASS | FS=0;MQ=199.84;MQRankSum=0;QD=7.03;ReadPosRankSum=0;SOR=0.693;FractionInformativeReads=0.667;DP=69;AF=0.5,0.5,0.5,0.5,0.5,0.5,0.5,0.5;AN=34;AC=6,7,2,6,1,3,1,1;OLD_MULTIALLELIC=chr1:97158472:GACACACACAC/GACACACACACACAC/GACACACACACAC/GACACACACACACACACACAC/GACACACACACACACACAC/G/GACACACACACACACAC/GACACACAC/GACACAC |
| chr1 | 97158472 | . | GACACACACAC | GACACACACACAC | 30.95 | PASS | FS=0;MQ=199.84;MQRankSum=0;QD=7.03;ReadPosRankSum=0;SOR=0.693;FractionInformativeReads=0.667;DP=69;AF=0.5,0.5,0.5,0.5,0.5,0.5,0.5,0.5;AN=34;AC=6,7,2,6,1,3,1,1;OLD_MULTIALLELIC=chr1:97158472:GACACACACAC/GACACACACACACAC/GACACACACACAC/GACACACACACACACACACAC/GACACACACACACACACAC/G/GACACACACACACACAC/GACACACAC/GACACAC |
| chr1 | 97158472 | . | GACACACACAC | GACACACACACACACACACAC | 30.95 | PASS | FS=0;MQ=199.84;MQRankSum=0;QD=7.03;ReadPosRankSum=0;SOR=0.693;FractionInformativeReads=0.667;DP=69;AF=0.5,0.5,0.5,0.5,0.5,0.5,0.5,0.5;AN=34;AC=6,7,2,6,1,3,1,1;OLD_MULTIALLELIC=chr1:97158472:GACACACACAC/GACACACACACACAC/GACACACACACAC/GACACACACACACACACACAC/GACACACACACACACACAC/G/GACACACACACACACAC/GACACACAC/GACACAC |
| chr1 | 97158472 | . | GACACACACAC | GACACACACACACACACAC | 30.95 | PASS | FS=0;MQ=199.84;MQRankSum=0;QD=7.03;ReadPosRankSum=0;SOR=0.693;FractionInformativeReads=0.667;DP=69;AF=0.5,0.5,0.5,0.5,0.5,0.5,0.5,0.5;AN=34;AC=6,7,2,6,1,3,1,1;OLD_MULTIALLELIC=chr1:97158472:GACACACACAC/GACACACACACACAC/GACACACACACAC/GACACACACACACACACACAC/GACACACACACACACACAC/G/GACACACACACACACAC/GACACACAC/GACACAC |
| chr1 | 97158472 | . | GACACACACAC | G | 30.95 | PASS | FS=0;MQ=199.84;MQRankSum=0;QD=7.03;ReadPosRankSum=0;SOR=0.693;FractionInformativeReads=0.667;DP=69;AF=0.5,0.5,0.5,0.5,0.5,0.5,0.5,0.5;AN=34;AC=6,7,2,6,1,3,1,1;OLD_MULTIALLELIC=chr1:97158472:GACACACACAC/GACACACACACACAC/GACACACACACAC/GACACACACACACACACACAC/GACACACACACACACACAC/G/GACACACACACACACAC/GACACACAC/GACACAC |
| chr1 | 97158472 | . | GACACACACAC | GACACACACACACACAC | 30.95 | PASS | FS=0;MQ=199.84;MQRankSum=0;QD=7.03;ReadPosRankSum=0;SOR=0.693;FractionInformativeReads=0.667;DP=69;AF=0.5,0.5,0.5,0.5,0.5,0.5,0.5,0.5;AN=34;AC=6,7,2,6,1,3,1,1;OLD_MULTIALLELIC=chr1:97158472:GACACACACAC/GACACACACACACAC/GACACACACACAC/GACACACACACACACACACAC/GACACACACACACACACAC/G/GACACACACACACACAC/GACACACAC/GACACAC |
| chr1 | 97158472 | . | GACACACACAC | GACACACAC | 30.95 | PASS | FS=0;MQ=199.84;MQRankSum=0;QD=7.03;ReadPosRankSum=0;SOR=0.693;FractionInformativeReads=0.667;DP=69;AF=0.5,0.5,0.5,0.5,0.5,0.5,0.5,0.5;AN=34;AC=6,7,2,6,1,3,1,1;OLD_MULTIALLELIC=chr1:97158472:GACACACACAC/GACACACACACACAC/GACACACACACAC/GACACACACACACACACACAC/GACACACACACACACACAC/G/GACACACACACACACAC/GACACACAC/GACACAC |
| chr1 | 97158472 | . | GACACACACAC | GACACAC | 30.95 | PASS | FS=0;MQ=199.84;MQRankSum=0;QD=7.03;ReadPosRankSum=0;SOR=0.693;FractionInformativeReads=0.667;DP=69;AF=0.5,0.5,0.5,0.5,0.5,0.5,0.5,0.5;AN=34;AC=6,7,2,6,1,3,1,1;OLD_MULTIALLELIC=chr1:97158472:GACACACACAC/GACACACACACACAC/GACACACACACAC/GACACACACACACACACACAC/GACACACACACACACACAC/G/GACACACACACACACAC/GACACACAC/GACACAC |
| chr1 | 97176868 | . | A | ATGTGTGTGTGTGTGTGTG | 28.92 | PASS | FS=0;MQ=196.7;MQRankSum=0;QD=27.39;ReadPosRankSum=0;SOR=0.693;FractionInformativeReads=1;DP=10;AF=0.5,0.5,0.5,0.5,0.5;AN=8;AC=2,1,1,1,1;OLD_MULTIALLELIC=chr1:97176868:A/ATGTGTGTGTGTGTGTGTG/ATGTG/ATGTGTGTGTGTGTGTGTGTG/ATGTGTG/ATGTGTGTGTGTGTG |
| chr1 | 97176868 | . | A | ATGTG | 28.92 | PASS | FS=0;MQ=196.7;MQRankSum=0;QD=27.39;ReadPosRankSum=0;SOR=0.693;FractionInformativeReads=1;DP=10;AF=0.5,0.5,0.5,0.5,0.5;AN=8;AC=2,1,1,1,1;OLD_MULTIALLELIC=chr1:97176868:A/ATGTGTGTGTGTGTGTGTG/ATGTG/ATGTGTGTGTGTGTGTGTGTG/ATGTGTG/ATGTGTGTGTGTGTG |
| chr1 | 97176868 | . | A | ATGTGTGTGTGTGTGTGTGTG | 28.92 | PASS | FS=0;MQ=196.7;MQRankSum=0;QD=27.39;ReadPosRankSum=0;SOR=0.693;FractionInformativeReads=1;DP=10;AF=0.5,0.5,0.5,0.5,0.5;AN=8;AC=2,1,1,1,1;OLD_MULTIALLELIC=chr1:97176868:A/ATGTGTGTGTGTGTGTGTG/ATGTG/ATGTGTGTGTGTGTGTGTGTG/ATGTGTG/ATGTGTGTGTGTGTG |
| chr1 | 97176868 | . | A | ATGTGTG | 28.92 | PASS | FS=0;MQ=196.7;MQRankSum=0;QD=27.39;ReadPosRankSum=0;SOR=0.693;FractionInformativeReads=1;DP=10;AF=0.5,0.5,0.5,0.5,0.5;AN=8;AC=2,1,1,1,1;OLD_MULTIALLELIC=chr1:97176868:A/ATGTGTGTGTGTGTGTGTG/ATGTG/ATGTGTGTGTGTGTGTGTGTG/ATGTGTG/ATGTGTGTGTGTGTG |
| chr1 | 97176868 | . | A | ATGTGTGTGTGTGTG | 28.92 | PASS | FS=0;MQ=196.7;MQRankSum=0;QD=27.39;ReadPosRankSum=0;SOR=0.693;FractionInformativeReads=1;DP=10;AF=0.5,0.5,0.5,0.5,0.5;AN=8;AC=2,1,1,1,1;OLD_MULTIALLELIC=chr1:97176868:A/ATGTGTGTGTGTGTGTGTG/ATGTG/ATGTGTGTGTGTGTGTGTGTG/ATGTGTG/ATGTGTGTGTGTGTG |
| chr1 | 97223388 | . | A | AACAC | 17.49 | PASS | FS=0;MQ=250;MQRankSum=0.804;QD=6.06;ReadPosRankSum=0.804;SOR=1.447;FractionInformativeReads=1;DP=4;AF=0.5,0.5;AN=4;AC=1,1;OLD_MULTIALLELIC=chr1:97223388:A/AACAC/AACACAC |
| chr1 | 97223388 | . | A | AACACAC | 17.49 | PASS | FS=0;MQ=250;MQRankSum=0.804;QD=6.06;ReadPosRankSum=0.804;SOR=1.447;FractionInformativeReads=1;DP=4;AF=0.5,0.5;AN=4;AC=1,1;OLD_MULTIALLELIC=chr1:97223388:A/AACAC/AACACAC |
| chr1 | 97335299 | . | TACACACACAC | TACACACACACAC | 12.83 | PASS | FS=0;MQ=199.58;MQRankSum=0;QD=9.71;ReadPosRankSum=0;SOR=0.693;FractionInformativeReads=1;DP=17;AF=0.5,0.5,0.5,0.5;AN=14;AC=4,1,1,5;OLD_MULTIALLELIC=chr1:97335299:TACACACACAC/TACACACACACAC/TACACACAC/T/TACACACACACACAC |
| chr1 | 97335299 | . | TACACACACAC | TACACACAC | 12.83 | PASS | FS=0;MQ=199.58;MQRankSum=0;QD=9.71;ReadPosRankSum=0;SOR=0.693;FractionInformativeReads=1;DP=17;AF=0.5,0.5,0.5,0.5;AN=14;AC=4,1,1,5;OLD_MULTIALLELIC=chr1:97335299:TACACACACAC/TACACACACACAC/TACACACAC/T/TACACACACACACAC |
| chr1 | 97335299 | . | TACACACACAC | T | 12.83 | PASS | FS=0;MQ=199.58;MQRankSum=0;QD=9.71;ReadPosRankSum=0;SOR=0.693;FractionInformativeReads=1;DP=17;AF=0.5,0.5,0.5,0.5;AN=14;AC=4,1,1,5;OLD_MULTIALLELIC=chr1:97335299:TACACACACAC/TACACACACACAC/TACACACAC/T/TACACACACACACAC |
| chr1 | 97335299 | . | TACACACACAC | TACACACACACACAC | 12.83 | PASS | FS=0;MQ=199.58;MQRankSum=0;QD=9.71;ReadPosRankSum=0;SOR=0.693;FractionInformativeReads=1;DP=17;AF=0.5,0.5,0.5,0.5;AN=14;AC=4,1,1,5;OLD_MULTIALLELIC=chr1:97335299:TACACACACAC/TACACACACACAC/TACACACAC/T/TACACACACACACAC |
| chr1 | 97525978 | . | A | AGTGTGT | 16.93 | PASS | FS=0;MQ=188.69;MQRankSum=0.731;QD=9.3;ReadPosRankSum=0.731;SOR=1.447;FractionInformativeReads=1;DP=5;AF=0.5,0.5;AN=2;AC=1,1;OLD_MULTIALLELIC=chr1:97525978:A/AGTGTGT/T |
| chr1 | 97525978 | . | A | T | 16.93 | PASS | FS=0;MQ=188.69;MQRankSum=0.731;QD=9.3;ReadPosRankSum=0.731;SOR=1.447;FractionInformativeReads=1;DP=5;AF=0.5,0.5;AN=2;AC=1,1;OLD_MULTIALLELIC=chr1:97525978:A/AGTGTGT/T |
| chr1 | 97525978 | . | AGTGT | AGTGTGTGTGTGT | 32.96 | PASS | FS=0;MQ=157;MQRankSum=0;QD=14.6;ReadPosRankSum=0;SOR=1.609;FractionInformativeReads=0.5;DP=16;AF=0.5,0.5,0.5,0.5,0.5,0.5,0.5,0.5;AN=16;AC=2,2,1,3,1,1,1,1;OLD_MULTIALLELIC=chr1:97525978:AGTGT/AGTGTGTGTGTGT/AGTGTGTGT/AGTGTGTGTGT/AGTGTGT/AGT/A/AGTGTGTGTGTGTGTGT/AGTGTGTGTGTGTGTGTGT |
| chr1 | 97525978 | . | AGTGT | AGTGTGTGT | 32.96 | PASS | FS=0;MQ=157;MQRankSum=0;QD=14.6;ReadPosRankSum=0;SOR=1.609;FractionInformativeReads=0.5;DP=16;AF=0.5,0.5,0.5,0.5,0.5,0.5,0.5,0.5;AN=16;AC=2,2,1,3,1,1,1,1;OLD_MULTIALLELIC=chr1:97525978:AGTGT/AGTGTGTGTGTGT/AGTGTGTGT/AGTGTGTGTGT/AGTGTGT/AGT/A/AGTGTGTGTGTGTGTGT/AGTGTGTGTGTGTGTGTGT |
| chr1 | 97525978 | . | AGTGT | AGTGTGTGTGT | 32.96 | PASS | FS=0;MQ=157;MQRankSum=0;QD=14.6;ReadPosRankSum=0;SOR=1.609;FractionInformativeReads=0.5;DP=16;AF=0.5,0.5,0.5,0.5,0.5,0.5,0.5,0.5;AN=16;AC=2,2,1,3,1,1,1,1;OLD_MULTIALLELIC=chr1:97525978:AGTGT/AGTGTGTGTGTGT/AGTGTGTGT/AGTGTGTGTGT/AGTGTGT/AGT/A/AGTGTGTGTGTGTGTGT/AGTGTGTGTGTGTGTGTGT |
| chr1 | 97525978 | . | AGTGT | AGTGTGT | 32.96 | PASS | FS=0;MQ=157;MQRankSum=0;QD=14.6;ReadPosRankSum=0;SOR=1.609;FractionInformativeReads=0.5;DP=16;AF=0.5,0.5,0.5,0.5,0.5,0.5,0.5,0.5;AN=16;AC=2,2,1,3,1,1,1,1;OLD_MULTIALLELIC=chr1:97525978:AGTGT/AGTGTGTGTGTGT/AGTGTGTGT/AGTGTGTGTGT/AGTGTGT/AGT/A/AGTGTGTGTGTGTGTGT/AGTGTGTGTGTGTGTGTGT |
| chr1 | 97525978 | . | AGTGT | AGT | 32.96 | PASS | FS=0;MQ=157;MQRankSum=0;QD=14.6;ReadPosRankSum=0;SOR=1.609;FractionInformativeReads=0.5;DP=16;AF=0.5,0.5,0.5,0.5,0.5,0.5,0.5,0.5;AN=16;AC=2,2,1,3,1,1,1,1;OLD_MULTIALLELIC=chr1:97525978:AGTGT/AGTGTGTGTGTGT/AGTGTGTGT/AGTGTGTGTGT/AGTGTGT/AGT/A/AGTGTGTGTGTGTGTGT/AGTGTGTGTGTGTGTGTGT |
| chr1 | 97525978 | . | AGTGT | A | 32.96 | PASS | FS=0;MQ=157;MQRankSum=0;QD=14.6;ReadPosRankSum=0;SOR=1.609;FractionInformativeReads=0.5;DP=16;AF=0.5,0.5,0.5,0.5,0.5,0.5,0.5,0.5;AN=16;AC=2,2,1,3,1,1,1,1;OLD_MULTIALLELIC=chr1:97525978:AGTGT/AGTGTGTGTGTGT/AGTGTGTGT/AGTGTGTGTGT/AGTGTGT/AGT/A/AGTGTGTGTGTGTGTGT/AGTGTGTGTGTGTGTGTGT |
| chr1 | 97525978 | . | AGTGT | AGTGTGTGTGTGTGTGT | 32.96 | PASS | FS=0;MQ=157;MQRankSum=0;QD=14.6;ReadPosRankSum=0;SOR=1.609;FractionInformativeReads=0.5;DP=16;AF=0.5,0.5,0.5,0.5,0.5,0.5,0.5,0.5;AN=16;AC=2,2,1,3,1,1,1,1;OLD_MULTIALLELIC=chr1:97525978:AGTGT/AGTGTGTGTGTGT/AGTGTGTGT/AGTGTGTGTGT/AGTGTGT/AGT/A/AGTGTGTGTGTGTGTGT/AGTGTGTGTGTGTGTGTGT |
| chr1 | 97525978 | . | AGTGT | AGTGTGTGTGTGTGTGTGT | 32.96 | PASS | FS=0;MQ=157;MQRankSum=0;QD=14.6;ReadPosRankSum=0;SOR=1.609;FractionInformativeReads=0.5;DP=16;AF=0.5,0.5,0.5,0.5,0.5,0.5,0.5,0.5;AN=16;AC=2,2,1,3,1,1,1,1;OLD_MULTIALLELIC=chr1:97525978:AGTGT/AGTGTGTGTGTGT/AGTGTGTGT/AGTGTGTGTGT/AGTGTGT/AGT/A/AGTGTGTGTGTGTGTGT/AGTGTGTGTGTGTGTGTGT |
| chr1 | 97724296 | . | GGGGGGGGGGGGGTGTGTGTGTGTGTGTGTGTGT | GGTGTGTGT | 8.87 | PASS | FS=0;MQ=144.39;MQRankSum=0.736;QD=9.17;ReadPosRankSum=0.736;SOR=0.368;FractionInformativeReads=1;DP=14;AF=0.5,1,1;AN=12;AC=3,2,5;OLD_MULTIALLELIC=chr1:97724296:GGGGGGGGGGGGGTGTGTGTGTGTGTGTGTGTGT/GGTGTGTGT/G/GGTGTGT |
| chr1 | 97724296 | . | GGGGGGGGGGGGGTGTGTGTGTGTGTGTGTGTGT | G | 8.87 | PASS | FS=0;MQ=144.39;MQRankSum=0.736;QD=9.17;ReadPosRankSum=0.736;SOR=0.368;FractionInformativeReads=1;DP=14;AF=0.5,1,1;AN=12;AC=3,2,5;OLD_MULTIALLELIC=chr1:97724296:GGGGGGGGGGGGGTGTGTGTGTGTGTGTGTGTGT/GGTGTGTGT/G/GGTGTGT |
| chr1 | 97724296 | . | GGGGGGGGGGGGGTGTGTGTGTGTGTGTGTGTGT | GGTGTGT | 8.87 | PASS | FS=0;MQ=144.39;MQRankSum=0.736;QD=9.17;ReadPosRankSum=0.736;SOR=0.368;FractionInformativeReads=1;DP=14;AF=0.5,1,1;AN=12;AC=3,2,5;OLD_MULTIALLELIC=chr1:97724296:GGGGGGGGGGGGGTGTGTGTGTGTGTGTGTGTGT/GGTGTGTGT/G/GGTGTGT |
| chr1 | 97736850 | . | T | TTG | 13.61 | PASS | FS=0;MQ=250;MQRankSum=0.731;QD=7.01;ReadPosRankSum=0.731;SOR=0.223;FractionInformativeReads=1;DP=15;AF=0.5,0.5,0.5;AN=10;AC=3,3,1;OLD_MULTIALLELIC=chr1:97736850:T/TTG/TTGTG/TTTG |
| chr1 | 97736850 | . | T | TTGTG | 13.61 | PASS | FS=0;MQ=250;MQRankSum=0.731;QD=7.01;ReadPosRankSum=0.731;SOR=0.223;FractionInformativeReads=1;DP=15;AF=0.5,0.5,0.5;AN=10;AC=3,3,1;OLD_MULTIALLELIC=chr1:97736850:T/TTG/TTGTG/TTTG |
| chr1 | 97736850 | . | T | TTTG | 13.61 | PASS | FS=0;MQ=250;MQRankSum=0.731;QD=7.01;ReadPosRankSum=0.731;SOR=0.223;FractionInformativeReads=1;DP=15;AF=0.5,0.5,0.5;AN=10;AC=3,3,1;OLD_MULTIALLELIC=chr1:97736850:T/TTG/TTGTG/TTTG |
| chr1 | 97813915 | . | GACACAC | GACACACAC | 9.79 | PASS | FS=0;MQ=177.05;MQRankSum=0;QD=6.15;ReadPosRankSum=0;SOR=2.303;FractionInformativeReads=1;DP=7;AF=1,0.5,0.5;AN=6;AC=4,1,1;OLD_MULTIALLELIC=chr1:97813915:GACACAC/GACACACAC/G/GAC |
| chr1 | 97813915 | . | GACACAC | G | 9.79 | PASS | FS=0;MQ=177.05;MQRankSum=0;QD=6.15;ReadPosRankSum=0;SOR=2.303;FractionInformativeReads=1;DP=7;AF=1,0.5,0.5;AN=6;AC=4,1,1;OLD_MULTIALLELIC=chr1:97813915:GACACAC/GACACACAC/G/GAC |
| chr1 | 97813915 | . | GACACAC | GAC | 9.79 | PASS | FS=0;MQ=177.05;MQRankSum=0;QD=6.15;ReadPosRankSum=0;SOR=2.303;FractionInformativeReads=1;DP=7;AF=1,0.5,0.5;AN=6;AC=4,1,1;OLD_MULTIALLELIC=chr1:97813915:GACACAC/GACACACAC/G/GAC |
| chr1 | 97832045 | . | T | TTGTGTGTGTG | 33.36 | PASS | FS=0;MQ=167.83;MQRankSum=0.727;QD=8.42;ReadPosRankSum=0.727;SOR=2.788;FractionInformativeReads=0.8;DP=49;AF=0.5,0.5,0.5,0.5,0.5,0.5,1;AN=34;AC=5,3,4,7,1,1,2;OLD_MULTIALLELIC=chr1:97832045:T/TTGTGTGTGTG/TTGTGTGTGTGTG/TTGTGTG/TTGTGTGTG/TTGTG/TTG/TTTTGTGTGTGTGTGTG |
| chr1 | 97832045 | . | T | TTGTGTGTGTGTG | 33.36 | PASS | FS=0;MQ=167.83;MQRankSum=0.727;QD=8.42;ReadPosRankSum=0.727;SOR=2.788;FractionInformativeReads=0.8;DP=49;AF=0.5,0.5,0.5,0.5,0.5,0.5,1;AN=34;AC=5,3,4,7,1,1,2;OLD_MULTIALLELIC=chr1:97832045:T/TTGTGTGTGTG/TTGTGTGTGTGTG/TTGTGTG/TTGTGTGTG/TTGTG/TTG/TTTTGTGTGTGTGTGTG |
| chr1 | 97832045 | . | T | TTGTGTG | 33.36 | PASS | FS=0;MQ=167.83;MQRankSum=0.727;QD=8.42;ReadPosRankSum=0.727;SOR=2.788;FractionInformativeReads=0.8;DP=49;AF=0.5,0.5,0.5,0.5,0.5,0.5,1;AN=34;AC=5,3,4,7,1,1,2;OLD_MULTIALLELIC=chr1:97832045:T/TTGTGTGTGTG/TTGTGTGTGTGTG/TTGTGTG/TTGTGTGTG/TTGTG/TTG/TTTTGTGTGTGTGTGTG |
| chr1 | 97832045 | . | T | TTGTGTGTG | 33.36 | PASS | FS=0;MQ=167.83;MQRankSum=0.727;QD=8.42;ReadPosRankSum=0.727;SOR=2.788;FractionInformativeReads=0.8;DP=49;AF=0.5,0.5,0.5,0.5,0.5,0.5,1;AN=34;AC=5,3,4,7,1,1,2;OLD_MULTIALLELIC=chr1:97832045:T/TTGTGTGTGTG/TTGTGTGTGTGTG/TTGTGTG/TTGTGTGTG/TTGTG/TTG/TTTTGTGTGTGTGTGTG |
| chr1 | 97832045 | . | T | TTGTG | 33.36 | PASS | FS=0;MQ=167.83;MQRankSum=0.727;QD=8.42;ReadPosRankSum=0.727;SOR=2.788;FractionInformativeReads=0.8;DP=49;AF=0.5,0.5,0.5,0.5,0.5,0.5,1;AN=34;AC=5,3,4,7,1,1,2;OLD_MULTIALLELIC=chr1:97832045:T/TTGTGTGTGTG/TTGTGTGTGTGTG/TTGTGTG/TTGTGTGTG/TTGTG/TTG/TTTTGTGTGTGTGTGTG |
| chr1 | 97832045 | . | T | TTG | 33.36 | PASS | FS=0;MQ=167.83;MQRankSum=0.727;QD=8.42;ReadPosRankSum=0.727;SOR=2.788;FractionInformativeReads=0.8;DP=49;AF=0.5,0.5,0.5,0.5,0.5,0.5,1;AN=34;AC=5,3,4,7,1,1,2;OLD_MULTIALLELIC=chr1:97832045:T/TTGTGTGTGTG/TTGTGTGTGTGTG/TTGTGTG/TTGTGTGTG/TTGTG/TTG/TTTTGTGTGTGTGTGTG |
| chr1 | 97832045 | . | T | TTTTGTGTGTGTGTGTG | 33.36 | PASS | FS=0;MQ=167.83;MQRankSum=0.727;QD=8.42;ReadPosRankSum=0.727;SOR=2.788;FractionInformativeReads=0.8;DP=49;AF=0.5,0.5,0.5,0.5,0.5,0.5,1;AN=34;AC=5,3,4,7,1,1,2;OLD_MULTIALLELIC=chr1:97832045:T/TTGTGTGTGTG/TTGTGTGTGTGTG/TTGTGTG/TTGTGTGTG/TTGTG/TTG/TTTTGTGTGTGTGTGTG |

**Supplementary table 6: Mapping table for DPYD variant, assigned activity score and annotated phenotype**

| Sr. No. | variant Id/detail | Exon and amino acid change | Genotype | Number of patients | Allele function category | Assigned Activity value (per variant allele) | Reference allele activity value, if any | Calculated activity score (for genotype) | Phenoptype as per CPIC annotations |
| --- | --- | --- | --- | --- | --- | --- | --- | --- | --- |
| 1 | rs112766203 | DPYD:NM_000110:exon18:c.C2279T:p.T760I | 0/1 | 1 | exonic | 0.5 | 1.0 | 1.5 | Intermediate Metabolizer |
| 2 | rs1801160 | DPYD:NM_000110:exon18:c.G2194A:p.V732I | 0/1 | 12 | exonic | 1.0 | 1.0 | 2.0 | Normal Metabolizer |
|  |  |  | 1/1 | 1 | exonic | 1.0 + 1.0 | - | 2.0 | Normal Metabolizer |
| 3 | rs17376848 | DPYD:NM_000110:exon14:c.T1896C:p.F632F | 0/1 | 8 | exonic | 1.0 | 1.0 | 2.0 | Normal Metabolizer |
| 4 | rs1801159 | DPYD:NM_000110:exon13:c.A1627G:p.I543V | 0/1 | 11 | exonic | 1.0 | 1.0 | 2.0 | Normal Metabolizer |
|  |  |  | 1/1 | 1 | exonic | 1.0 + 1.0 | - | 2.0 | Normal Metabolizer |
| 5 | rs1801158 | DPYD:NM_000110:exon13:c.G1601A:p.S534N | 0/1 | 1 | exonic | 1.0 | 1.0 | 2.0 | Normal Metabolizer |
| 6 | . | DPYD:NM_000110:exon13:c.T1545G:p.V515V | 0/1 | 1 | exonic | No information | 1.0 | No information | No information |
| 7 | rs148994843 | DPYD:NM_000110:exon13:c.G1543A:p.V515I | 0/1 | 2 | exonic | 1.0 | 1.0 | 2.0 | Normal Metabolizer |
| 8 | rs56038477 | DPYD:NM_000110:exon11:c.G1236A:p.E412E | 0/1 | 1 | exonic | 0.5 | 1.0 | 1.5 | Intermediate Metabolizer |
| 9 | rs45589337 | DPYD:NM_000110:exon8:c.A775G:p.K259E | 0/1 | 1 | exonic | 1.0 | 1.0 | 2.0 | Normal Metabolizer |
| 10 | rs2297595 | DPYD:NM_000110:exon6:c.A496G:p.M166V | 0/1 | 7 | exonic | 1.0 | 1.0 | 2.0 | Normal Metabolizer |
| 11 | rs771573678 | DPYD:NM_000110:exon4:c.A287T:p.D96V,DPYD:NM_001160301:exon4:c.A287T:p.D96V | 0/1 | 1 | exonic | No information | 1.0 | No information | No information |
| 12 | rs1801265 | DPYD:NM_000110:exon2:c.T85C:p.C29R,DPYD:NM_001160301:exon2:c.T85C:p.C29R | 0/1 | 25 | exonic | 1.0 | 1.0 | 2.0 | Normal Metabolizer |
|  |  |  | 1/1 | 9 | exonic | 1.0 + 1.0 | - | 2.0 | Normal Metabolizer |
| 13 | rs3918290 | . | 0/1 | 1 | Splicing | 0.0 | 1.0 | 1.0 | Intermediate Metabolizer |

**Supplementary table 7: Correlation of toxicity and mutations observed in patients**

|  | **DPYD VARIANT** | **DPYD WILD TYPE** |
| --- | --- | --- |
| **Overall**  Grade 1  2  3 | 37 (68.5%)  1(1.9%)  27(50%)  9(16.7%) | 15(68.1%)  1(4.5%)  9(40.9%)  5(22.7%) |
| **HFS**  Gr1  Gr2  Gr3 | 7(13%)  1(1.9%)  5(9.3%)  1(1.9%) | 3(13.5%)  1(4.5%)  1(4.5%)  1(4.5%) |
| **Diarrhoea**  Gr 1  Gr 2  Gr 3 | 22(40.8)  1(1.9%)  14(25.9)  7(13%) | 10(45.4)  -  5(22.7)  5(22.7) |
| **Mucositis**  Gr1  Gr2  Gr3 | 22(40.8)  1(1.9)  19(35.2)  2(3.7) | 7(31.7%)  1(4.5)  5(22.7)  1(4.5%) |
| **Haematological**  Gr2 | 2(3.7%) | 1(4.5%) |

**Supplementary table 8: Variant specific genotype and observed toxicity in our cohort**

| **Sr. No.** | **Variant ID** | **Exon and amino acid change** | **Genotype** | **Number of patients** | **Observed toxicity grade ≥ 3 in our patients** | **Matched Phenotype as per CPIC guidelines** |
| --- | --- | --- | --- | --- | --- | --- |
| 1 | rs112766203 | DPYD:NM_000110:exon18:c.C2279T:p.T760I | 0/1 | 1 | NA | 1.5 (Intermediate Metabolizer) |
| 2 | rs1801160 | DPYD:NM_000110:exon18:c.G2194A:p.V732I | 0/1 | 12 | Diarrhea grade 3 (n=3)  Mucositis grade 3 (n=1)  Hand Foot Syndrome grade 3 + Mucositis grade 2 (n=1) | 2.0 (Normal Metabolizer) |
|  |  |  | 1/1 | 1 | NA | 2.0 (Normal Metabolizer) |
| 3 | rs17376848 | DPYD:NM_000110:exon14:c.T1896C:p.F632F | 0/1 | 8 | Diarrhea grade 3 + Mucositis grade 2 (n=1) | 2.0 (Normal Metabolizer) |
| 4 | rs1801159 | DPYD:NM_000110:exon13:c.A1627G:p.I543V | 0/1 | 11 | Diarrhea grade 3 (n=1)  Mucositis grade 3 (n=1)  Hand Foot Syndrome grade 3 + Mucositis grade 2 (n=1) | 2.0 (Normal Metabolizer) |
|  |  |  | 1/1 | 1 | NA | 2.0 (Normal Metabolizer) |
| 5 | rs1801158 | DPYD:NM_000110:exon13:c.G1601A:p.S534N | 0/1 | 1 | NA | 2.0 (Normal Metabolizer) |
| 6 | . | DPYD:NM_000110:exon13:c.T1545G:p.V515V | 0/1 | 1 | NA | No information |
| 7 | rs148994843 | DPYD:NM_000110:exon13:c.G1543A:p.V515I | 0/1 | 2 | Diarrhea grade 3 (n=1) | 2.0 (Normal Metabolizer) |
| 8 | rs56038477 | DPYD:NM_000110:exon11:c.G1236A:p.E412E | 0/1 | 1 | NA | 1.5 (Intermediate Metabolizer) |
| 9 | rs45589337 | DPYD:NM_000110:exon8:c.A775G:p.K259E | 0/1 | 1 | Diarrhea grade 2, Mucositis grade 3 (n=1) | 2.0 (Normal Metabolizer) |
| 10 | rs2297595 | DPYD:NM_000110:exon6:c.A496G:p.M166V | 0/1 | 7 | Diarrhea grade 3 (n=1) | 2.0 (Normal Metabolizer) |
| 11 | rs771573678 | DPYD:NM_000110:exon4:c.A287T:p.D96V,DPYD:NM_001160301:exon4:c.A287T:p.D96V | 0/1 | 1 | NA | No information |
| 12 | rs1801265 | DPYD:NM_000110:exon2:c.T85C:p.C29R,DPYD:NM_001160301:exon2:c.T85C:p.C29R | 0/1 | 25 | Diarrhea grade 3 (n=2)  Mucositis grade 3 (n=1)  Hand Foot Syndrome grade 3 + Mucositis grade 2 (n=1)  Diarrhea grade 2 + Mucositis grade 3 (n=1)  Diarrhea grade 3 + Mucositis grade 2 (n=1) | 2.0 (Normal Metabolizer) |
|  |  |  | 1/1 | 9 | NA | 2.0 (Normal Metabolizer) |
| 13 | rs3918290 | . | 0/1 | 1 | Diarrhea grade 3 (n=1) | 1.0 (Intermediate Metabolizer) |

**Supplementary table 9: Toxicity Profile by DPYD Metabolizer Status**

| **Toxicity** | **OR** | **95% CI** | **P value** | **Fitted Probability (IM)** |
| --- | --- | --- | --- | --- |
| HFS G1/4 | 0.83 | 0.01–9.94 | ne | 0.902 |
| HFS Grade2/3 | 0.98 | 0.01–11.98 | NE | 0.989 |
| DIARRHOEA>GRADE2 | 1.15 | 0.1–9.33 | NE | 0.898 |
| DIARRHOEA ANY GRADE | 1.05 | 0.09–8.54 | NE | 0.961 |
| Mucositis any grade | 1.05 | 0.09–8.54 | NE | 0.961 |
| Mucositis grade>2 | 1.25 | 0.11–10.22 | NE | 0.834 |
| MYELO >GRADE2 | 2.77 | 0.02–44.91 | NE | 0.573 |
| MYELO ANY GRADE | 2.77 | 0.02–44.91 | NE | 0.573 |
| OVERALL >GR2 | 1.42 | 0.18–16.37 | NE | 0.738 |
| OVERALL ANY GRADE | 1.21 | 0.15–13.97 | NE | 0.855 |
| NE- Not estimable; IM- Intermediate metaboliser.  Analyses were performed using the logistf package in R. | | | | |
